# On the Utilization and Characterization of External Biotransformation Systems in *In Vitro* Toxicology: A Critical Review of the Scientific Literature with Guidance Recommendations

**DOI:** 10.1021/acsenvironau.5c00096

**Published:** 2025-11-27

**Authors:** Sebastian Lungu-Mitea, Matilda Stein Åslund, Inska Reichstein, Felipe Augusto Pinto-Vidal, Andreas Schiwy, Henner Hollert, Miriam N Jacobs, Klára Hilscherová

**Affiliations:** 1 Faculty of Science, RECETOX, 37748 Masaryk University, Brno 625 00, Czech Republic; 2 Department of Pharmaceutical Biosciences, Toxicology and Drug Safety, Uppsala University, Uppsala 751 24, Sweden; 3 Department of Biology and Environmental Science, Linnaeus University, Kalmar 392 31, Sweden; 4 Department of Forest Mycology and Plant Pathology, 8095 Swedish University of Agricultural Sciences, Uppsala 756 51, Sweden; 5 Department of Evolutionary Ecology and Environmental Toxicology, 9173 Goethe University Frankfurt, Frankfurt am Main 60438, Germany; 6 Department of Environmental Media Related Ecotoxicology, Fraunhofer Institute for Molecular Biology and Applied Ecology (FhG-IME), Schmallenberg 57392, Germany; 7 UK Health Security Agency, Radiation, Chemical, Climate and Environmental Hazards (RCCE), Harwell Science and Innovation Campus, Chilton OX11 0RQ OXON, U.K.; 8 Department of Animal Biosciences, 8095 Swedish University of Agricultural Sciences, Uppsala 756 51, Sweden

**Keywords:** biotransformation, S9, microsomes, *in vitro*, critical review, endocrine disruption, mutagenicity, genotoxicity

## Abstract

Incorporating biotransformation capabilities into *in vitro* assays represents one of the most critical challenges in toxicology, facilitating the transition from *in vivo* models to integrated *in vitro* strategies. Although emerging technologies show promise, their current limitations in scalability hinder their high-throughput applications. In the short to mid term, externally added biotransformation systems (“BTS”: S9 and microsomal liver fractions) used together with *in vitro* assays offer viable alternatives. However, despite over 50 years of use, BTS are marred by reproducibility issues, raising concerns about their reliability and raising the question: Are BTS inherently unreliable, or has their reputation been flawed by methodological oversights? This review critically evaluates BTS’ methodological rigor, applying a deep statistical analysis of the scientific literature. We employed Boolean operator searches across scientific literature repositories to curate a database on BTS research in conjunction with relevant *in vitro* assays, focusing on endocrine disruption, mutagenicity, and genotoxicity end points. Through systematic searches, screening, and eligibility criteria, we identified 229 bibliographic records. Data parametrization and extraction were conducted across 24 domains of BTS relevance and reliability. Methodological reporting rigor was assessed via scoring (reported vs nonreported data items) and revealed a lack of reproducible standards. Numerical measures associated with principal BTS reaction components were subjected to meta-regression analyses. Within the aggregated data set, no statistically significant correlations were found for BTS and related cofactor concentration–response relationships or time-related elements. Finally, descriptive statistics, multiple correspondence analysis, and *Apriori* algorithm-based relational networks identified qualitative patterns of methodological reporting robustness and deficiencies. In conclusion, these results emphasize shortcomings across the scientific literature in complying with appropriate methodological reporting. We offer evidence-based recommendations, in the form of a conceptual regulatory guidance framework, to enhance research practices, quality, and reproducibility of BTS applications, designed to strengthen the robustness of BTS research and its integration into regulatory-relevant hazard and risk assessment of chemicals.

## Graphical abstract

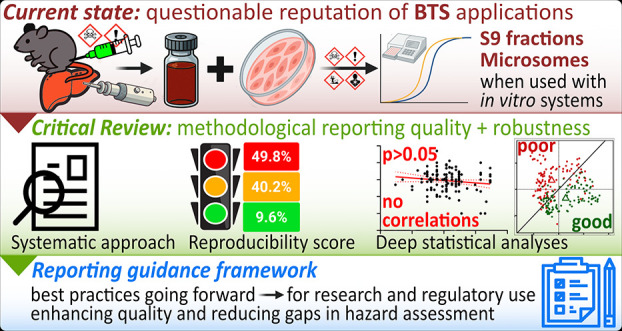

## 1 Introduction

### 1.1 Rationale

Over the past two decades, the focus of toxicological chemical hazard assessment has transitioned from relying primarily on *in vivo* animal lethality-based end points to embracing a mode of action and mechanistic methodology.^1–4^ Contextually, the incorporation of alternatives to *in vivo* animal testing has now become central to regulatory toxicology with the intention of ultimately replacing animal testing. Current European and national strategies aimed at reducing the risks posed by hazardous chemicals to health and the environment seek to minimize animal testing and implement strategies for next-generation risk assessment. In 2023, the EU Commission published a roadmap to achieve this.^5^ While *in vitro* test methods can be utilized to shed light on toxicological mechanisms, the inclusion of metabolism into *in vitro* testing strategies should be considered in a model-dependent manner, as *in vitro* methods are not a one-to-one replacement for the *in vivo* animal test methods. Combinations of *in vitro* test methods are needed to address end points of concern adequately.

Furthermore, a common limitation of many *in vitro* systems, as used in regulatory safety assessment and reported in the literature, is that they often lack, have reduced, or display altered biotransformation capacity compared to their tissue of origin.^6–9^ Some *in vitro* test systems, such as enzymatic assays or neuronal cultures, completely lack biotransformation capacity. Consequently, metabolism is not sufficiently addressed,^9^ as the xenobiotic metabolism machinery can transform parent compounds into more toxic (bioactivation) or less toxic (detoxification) metabolites.^7,10,11^ Therefore, such test systems may require metabolic substitution. Others, such as hepatocyte-derived cell lines, retain a certain level of metabolic activity under prolonged culture passage conditions, which may be sufficient to address xenobiotic metabolism in specific testing scenarios.^12–14^

To date, metabolizing systems have been routinely applied to standardized *in vitro* test guidelines (TGs) in relation to genotoxicity and mutagenicity, the *in vitro* micronucleus test,^15^ and the bacterial reverse mutation test, or Ames test.^16^ As such, these standardized test methods are routinely requested and relied upon for most industrial chemical, pharmaceutical, and agrochemical hazard testing. Furthermore, OECD TGs do recommend that ″exogenous metabolizing systems should be used in cell systems without metabolic capacity”.

In the early 2000s, efforts to review and improve the metabolic status of *in vitro* systems were undertaken by the OECD,^7–9^ with short-, medium-, and long-term recommendations. Short- to medium-term recommendations included the addition of noncytotoxic rodent liver tissue fractions such as microsomal systems and supernatants after 9000*g* centrifugation of liver homogenate (S9), containing phase 1 and phase 2 metabolic enzymes, i.e., cytochrome P450 (CYP450), uridine-diphosphate-glucuronosyltransferase (UGT), and cytosolic enzymes (biotransformation systems, hereafter “BTS”). (Notably, while we use BTS as an umbrella term for S9 and microsomal fractions, the term “final BTS reaction mixture” refers to the combination of BTS, cofactors, potentially applied cofactor regeneration systems, buffers, solvents, and exposure chemicals. This definition is nonexhaustive, as specific test setups may incorporate alternative reagents.)

The longer-term recommendations advocated for the development of a relevant cell system, such as the HepaRG CYP induction test method and the use of cryopreserved human hepatocytes. These have progressed, are discussed in, e.g.,^17,18^ and are not part of the work described here.

The International Council for Harmonisation of Technical Requirements for Pharmaceuticals for Human Use (ICH) has recently published the M12 Guideline, which provides specific and detailed guidance regarding the application of various hepatic *in vitro* systems and considerations regarding the application of Physiologically Based Pharmacokinetic (PBPK) computational modeling for drug interaction studies.^19^ The ICH guidance, however, is designed for the safety assessment of pharmaceuticals that will be entering clinical trials, while for chemical testing and hazard assessment purposes, the objective is not to ensure the safety and efficacy of a pharmaceutical intended for human consumption but to characterize the intrinsic hazards of the chemical as a protection and prevention measure in the design, application, risk assessment, and management of a chemical.

Other complementary research efforts include developing test systems that enhance biotransformation capabilities, such as some types of 3D cell cultures and microfluidic chips, although they are not yet ready for regulatory applications or high-throughput screening (HTS).^20,21^ The US EPA is exploring metabolically active tips to add to HTS cellular systems and has included metabolic assessment of cell line systems in Toxcast.^22,23^ Additional approaches include the stable or transient augmentation of microorganisms or cell lines via a genetic modification to include or enhance metabolic enzymes.^24–26^ Some of these approaches are not recent^27^ and have not gained broad application or regulatory acceptance.

BTS, as they are derived from animals, represent an *ex vivo* refinement rather than a complete replacement of *in vivo* methods. Nonetheless, their utility is high, evidenced by their well-established applications in biomedicine, pharmacology, toxicology, and other fields. This is supported by over a century of historical use and experience.^28,29^ Indeed, many protocols, including BTS, are available within an *in vitro* setup. Furthermore, S9 and microsomal BTS sourced from various vendors cover diverse species and configurations, including those obtained from chemically induced animals for enhanced BTS activity. (Historically, increased BTS-related metabolic capacity was induced into model test species by dietary exposure to mixtures of polychlorinated biphenyls (PCBs) known as “Aroclors”, particularly the Aroclor 1254 variant. However, following the phase-out of PCBs under the Stockholm Convention on Persistent Organic Pollutants,^30^ mixtures of β-naphthoflavone and phenobarbital (BNF/PB) have been utilized as substitutes).

BTS definition and variability are recognized as the major drawbacks for their integration into combinations with *in vitro* systems, including HTS applications. Common rodent-derived BTS often lack comprehensive characterization of vital aspects affecting their biotransformation capacities, such as specifications of BTS preparation, BTS protein concentration, enzyme activity, and experimental conditions used in the assay.^6^ This information is essential for accurately categorized and utilizing historical data. Prospectively, precise characterization and categorization of BTS are crucial to ensuring the quality, reproducibility, and comparability of their *in vitro* applications, especially for chemical biotransformation. Current BTS manufacturing protocols are often based upon procedures that have been in long-term use,^31–33^ but the new ICH guidance^19^ provides more contemporary quality criteria. Notably, many scientific literature studies have used self-manufactured BTS without explicitly detailing production protocols, and greater transparency in the production process is needed from commercial manufacturers to understand the inherent uncertainties when interpreting the resultant data. Awareness of variance and variability in BTS has been acknowledged for over 40 years,^34–38^ and in practice, a reasonably substantial amount of data using BTS with the Ames and micronucleus test methods is included in, e.g., agrochemical study report submissions to regulatory bodies.

Considering the ultimate objective of enhancing the human and environmental relevance of *in vitro* methodologies by incorporating metabolic components and facilitating scalable, reproducible HTS methods, we conducted a focused examination of the hazard assessment requirements for BTS to attain a regulatory-acceptable quality level. This entails ensuring adequate, consistent reporting characterization of BTS to guarantee reliability, relevance, and reproducibility. As pointed out above, previous detailed reviews and communications^6–8^ have pinpointed the deficiencies in BTS utilization, recommending that these issues be addressed. Yet, these recommendations have been largely overlooked in applied research. This review critically evaluates the existing scientific literature, focusing on the BTS methodological reporting rigor, by providing complex data and subsequent analysis.

### 1.2 Objectives

The overarching goal of this review is to evaluate the reporting standards of BTS within the existing scientific literature. This entails a meta-analysis of the relevant information on BTS and cofactor formulations, their concentrations, and reaction conditions (in the sense of chemical kinetics). Considerations for the development of a research and regulatory-relevant guidance framework to establish specific reporting criteria for BTS applications in conjunction with *in vitro* assays for chemical hazard assessment are provided. It is intended to complement the new ICH pharmaceutical guidelines.^19^ The purpose is to enhance the quality of BTS applications in *in vitro* regulatory contexts, including HTS.

Given BTS’ diverse origins and applications across a wide array of chemical compounds, this review is focused on the application of BTS to *in vitro* test systems for the detection of mutagens, genotoxicants, and endocrine active substances/disruptors, such as in, e.g., reporter-gene assays. Notably, these scope limitations were defined based on the prominence of the selected end points in toxicity testing involving BTS, the consequential numerical availability of relevant studies, their extensive coverage in TGs and guidance documents,^8,15,16,39,40^ and practical constraints related to literature search. Since the key determinant is the appropriate handling and reporting of BTS, which is transferable to the testing of other end points in diverse *in vitro* assays, we consider the results of our study to be broadly applicable across toxicological domains.

Next to the most commonly used rat-derived BTS, the review also encompasses human^41^ and fish-derived BTS. The primary focus is on BTS’ implementation and methodological reporting. This is achieved by evaluating and grading three primary domains (“BTS characterization”, “BTS reaction components”, and “BTS-specific experimental setup”) and 24 subdomains within the selected literature. Both quantitative and qualitative meta-analyses were conducted on the gathered data. Following analysis and synthesis, quality criteria are proposed to support researchers in adequate BTS parameter reporting, thereby improving their experimental designs and enhancing the utility and applicability of their BTS experiments in assessing toxicological metabolic hazards.

In summary, this review:

A. critically assesses the scientific rigor of BTS reporting standards in the published, peer-reviewed literature, focusing on the reproducibility, reliability, and robustness of the methodologies used via a grading scheme approach.
B. deconstructs and interprets concentration–response and reaction conditions of BTS protein and cofactor concentrations as reported in the literature.
C. investigates the extracted data for additional patterns, correlations, and identification of knowledge gaps.
D. formulates a guidance framework for future BTS applications in chemical hazard assessment, targeting regulatory and Test Guideline applications.

## 2 Methods

The critical review follows a systematic approach by employing tools for framing, scoping, eligibility, literature search, and screening, as utilized in systematic reviews (SR^42,43^ ), systematic evidence maps (SEM^44,45^ ), and scoping reviews (ScR^46^), with specific modifications toward the field of toxicology.^47–49^ Due to the nature of our investigation, we could not comply with canonical SR, SEM, or ScR outcomes.^50,51^ Instead, we conceptualized our structural framework to fit the specific needs (Figure S1 in the Supplementary Manuscript (SM)). The SM provides further details in Sections 2.4, 2.11, and 2.15. Previously, a review protocol was conceptualized, published, and shared among peers according to the PRISMA-P guidelines.^52,53^ The latter was uploaded to Figshare^54^ (10.6084/m9.figshare.21494616.v2, *accessed 2025/10/17*).

This section highlights the most critical aspects. For a more detailed insight into the applied methods and techniques, please refer to the SM, Section 2, which presents the materials and methods in a PRISMA-aligned manner and provides further details for all sections elaborated below. All raw data and metadata are provided as additional supplementary information materials (SI) S1–S11 and are available on Figshare (10.6084/m9.figshare.30257470, *accessed 2025/10/17*).^55^

This review did not include gray literature nor JMPR and EFSA/ECHA summary reports on agrochemicals and industrial chemicals.

### 2.1 Study Eligibility Criteria

Limitations: Peer-reviewed, English, and scientific literature. Only original scientific articles; no meta-analyses or review articles. No limitations: articles’ geographic origin or publication date. For more details, eligibility criteria reasoning, and explanations, see the SM, Section 2.3. PICO/PECO­(TS) criteria (population, intervention, exposure, comparator, outcomes, target conditions, study design) are described in Sections 2.4 and 2.9 of the SM.

### 2.2 Information Sources

Standard scientific literature repositories were employed to construct an information collection database using Boolean operator searches detailed below in Section 2.3 and the SM, Section 2.6. These repositories include PubMed (https://pubmed.ncbi.nlm.nih.gov/, *accessed 2025/10/17*), Web of Science (https://www.webofscience.com/, *accessed 2025/10/17*), and Scopus (https://www.scopus.com/, *accessed 2025/10/17*).

The search strategy was refined, and data item domains were coded by using a “piloting” process. A subset of 73 highly relevant publications previously identified by the reviewers (see SI5 and the reference list “BTS3” in the SM, Section 5.4) was initially screened and utilized for this purpose. Potential study biases, including those related to the selection of bibliographic records previously known to the authors, are addressed in Section 4.1 of the SM. For more details on the piloting process, see the SM, Section 2.5. The coding of data items is detailed in Section 2.6.

### 2.3 Search Strategy

The named repositories were searched using the following syntax and operators:

Search string 1: ″biotransformation″ AND ″metab*″ AND ″S9″ AND (″endocrine″ OR ″thyroid″ OR ″*estro*″ OR ″andro*″
Search string 2: ″biotransformation″ AND ″metab*″ AND ″S9″ AND ″cells″ AND ″cytotox*″ AND (″genotox*″ OR ″mutagen*″)

The main article, the SM, and supporting supplementary information material files (SI) refer to the databases and resulting bibliographies from the search strings as “BTS1/endocrine” and “BTS2/mutagen”. The search parameters were specifically tailored to focus on BTS implementation in *in vitro* studies addressing mutagenic, genotoxic, and endocrine-disrupting end points, to align with the objectives outlined in Section 1.2, and to maintain a manageable scope of search results. More details on Boolean operator specifics, search strategy reasoning, and search timelines are given in the SM, Section 2.6.

### 2.4 Selection of Studies

The search results from the specified literature repositories were downloaded and imported into the Sysrev GUI (https://sysrev.com/, *accessed 2025/10/17*) using PubMed identifiers (PMID). All search outputs were consolidated into a single database within Sysrev. Four independent reviewers employed this platform for article selection, using an article sequence randomization approach. The reviewers screened titles and abstracts based on the pre-established eligibility criteria in Section 2.1 and the SM, Section 2.3. Article inclusion was based on reviewer consensus with a 75% agreement threshold. For more details on study selection, bibliographic file formatting, and duplicate removal, consult the SM, Section 2.7.

### 2.5 Data Collection

Post-selection, the revised bibliography underwent a second round of manual duplicate removal, and full-text PDFs were retrieved.

Data extraction and evaluation adhered to the DEERS protocol (Data Extraction, Evaluation, and Reliability Schema; see the SM, Appendix 6.1 and Section 2.11). Each article’s DEERS information extracts were classified, organized, and summarized in a Microsoft Excel spreadsheet using a predefined extraction form for each reviewer (details in SI6). A unique identification number was allocated to each article, ensuring comprehensive data collection across all primary and secondary data domains.

Two reviewers independently conducted detailed data extraction. Four independent reviewers then cross-verified and refined the entire extraction data set. For more details on the used data formats, software, and data extraction, refer to the SM, Section 2.8.

### 2.6 Data Items and Coding

The coding for data items was established through a preliminary evaluation of 73 studies (part of “BTS3/historical”) deemed highly relevant (“piloting”) while referencing prior publications on key BTS experimental parameters.^6,8,9,21,56^ This process involved discussions and consensus among four reviewers to determine the coding strategy. In total, 24 domains were identified as essential for a comprehensive evaluation of the studies’ methodology, reporting, and reliability standards (Table 1). Of these 24 domains, nine were consistently detailed in all piloting studies and were considered significant for assessing study relevance but not for evaluating reliability. These nine domains are referred to as “data items of relevance” and are excluded from the reliability assessment (see also SM, Table S2, delineated as “non-critical”). The remaining 15 domains were deemed vital for methodological reliability assessment (outcome A; see also SM, Table S2, delineated as “critical”). The data items discussed in this section align with the criteria outlined in the DEERS protocol (refer to SM, Appendix 6.1). Methodological data item measures for four primary and 24 subdomains were extracted, as summarized in Table 1. For more information on the data item domain measures, consult the SM, Section 2.9. Table S2, within the SM, also provides a hypothetical example of data extraction following the established coding parameters.

**Table 1:** Total Data Item Domains Extracted from the Bibliography Database

**primary domains**	**subdomains**
BTS characterization	origin, species, strain, pooling (and sex), husbandry, and (xeno-)metabolic induction
BTS reaction components	total protein concentration, buffer system, dilution factor, primary cofactors, secondary cofactors, and used solvent
BTS experimental setup	incubation period, incubation temperature, and BTS-related controls
data items of relevance	author, year, journal, test system, end point, experimental methodology, type of applied external BTS, exposure, and post-BTS procedure

### 2.7 Outcomes, Effect Measures, and Synthesis Methods

We employed various effect measures and synthesis methods for each outcome based on the prioritization hierarchy outlined in Section 1.2, “Objectives”, and the SM, Section 2.10, “Outcomes and prioritisation”. Each primary study outcome (A–D) is aligned with the study objectives listed in Section 1.2. Outcomes A to C are assessed via the meta-analyses of extracted data item measures. In contrast, the synthesis of the latter directs outcome D (regulatory and research guidance framework) (see also Figure S1 for the structural framework of the critical review).

For outcome A (“scoring”), which focused on assessing BTS methodological reporting standards and scientific rigor as per the DEERS protocol, data items were scored binary (reported vs nonreported), resulting in a net score of one for each reported subdomain. This measure was applied to qualitative or quantitative data item domains (SM, Table S2 and SI6). We only scored critical data item domains for reliability (SM, Table S2), with 16 scoring categories derived from 15 critical domains, including the bifurcated “BTS pooling” domain. Each reported data item received a positive score of 1, whereas a nonreported item received a neutral score of 0. Data was manually entered into the DEERS protocol and transferred to a Microsoft Excel sheet (SI6) for summarization in a queryable table format database. Details on nonreported data items, visualization of scoring outcomes, and statistical analysis are provided in the SM, Section 2.12.

For the secondary study outcome B (“meta-regression”), we aimed to meta-analyze quantitative data item domain measures to identify concentration–response or reaction condition-related patterns in BTS applications. The parameters related to BTS, including the incubation period, BTS protein concentration, and primary cofactor concentration, exhibit hypothetical interdependence. For instance, an increase in the BTS protein concentration can provide a reduction in the incubation time. Consequently, we hypothesized that the relationship between these parameters should be characterized by either a linear or exponential correlation. Data item measures were log-transformed, checked for the fulfillment of parametric test criteria (see the SM, Section 2.12), and analyzed through simple linear regressions to derive adjusted *R* ^2^ values for model accuracy. Pearson’s correlation was used to determine the coefficients of correlation and determination. Additionally, paired comparisons were employed where quantitative data item measures were matched from the exact origin of the article record, as well as hand-curated subdata sets, which limited the comparison to phase- or substrate-specific kinetic effects. We conducted computations, statistical analyses, and graphical plotting in GraphPad Prism 8. For more methodological details, consult the SM, Section 2.12.

As a tertiary study outcome, outcome C (“mapping”) involved the exploratory mapping of all qualitative data item (sub)­domain measures. We simplified these domain measures for machine readability (see the SM, Table S4, and SI9) and analyzed them using descriptive and summary statistics, multiple factor correspondence analyses (MCA), and association networks (*Apriori* algorithms). For brevity, the following paragraphs summarize the utilized mapping analyses. More details are provided in the SM, Section 2.12.

Descriptive and summary statistics, including histograms, Euler plots, and Upset plots, were generated in R Studio^57^ and R software version 4.1.2,^58^ utilizing the R packages ggplot2, eulerr, and UpSetR.^59–61^

MCA was performed using FactoMineR^62^ in R Studio. The analysis included all qualitative data item subdomains from the simplified coding data set (see the SM, Table S4 and SI9) as active variables, which were transformed into a binary information matrix (for details, the SM, Section 2.12 and SI9), except for “publication year” (grouped in 5-year intervals), “publication group” (extraction data sets BTS1 to 3), and total scores, which were defined as qualitative supplementary variables and did not influence the analysis mathematically. Visualization in ggplot2 ^61^ was restricted to levels with more than five relative observations, with data ellipses representing normal probability contours at a 0.9 confidence level.

Lastly, for data association and relational networks, we utilized association rule mining with the *Apriori* algorithm in the arules package^63^ in R Studio to identify relational networks among qualitative data item subdomains in the simplified coding data set (see the SM, Table S4, and SI9). This process involved setting a support threshold of 10% and a confidence threshold of 80%, with visualization aided by the arulesviz package.^64^

Data item subdomains that exhibited robustness markers, either positively or negatively (see the SM, Table S9), in both MCA and *Apriori* frameworks, were further analyzed (confirmatory analyses^65^) to evaluate the effect sizes in different scoring subpopulations. For groups comprising two scoring subpopulations, nonparametric, two-sided Mann–Whitney *U* tests were employed (alpha level = 0.05). In multiple comparisons, the Kruskal–Wallis test was utilized as the primary analytical tool, followed by Dunn’s post hoc test (alpha level = 0.05). All of the mentioned tests were performed with GraphPad Prism 8.

## 3 Results and Discussion

Our comprehensive analysis, detailed in the subsequent sections and further elaborated in the supplementary manuscript (SM), revealed that the literature generally fell short of reaching adequate methodological reporting quality that would ensure reproducibility and downstream data utilization. Notably, throughout Section 3, we primarily evaluate the reporting quality of the identified data items and their respective measures, categorizing them as either “(methodologically) robust/sound” or “(methodologically) unsound/limited”, depending on the quality of reporting within specific records or clusters in the meta-analyses. This categorization does not pertain to the technical or functional performance of the documented BTS but rather to the reproducibility of their respective testing scenarios. As will be elaborated later, patterns observed in the iterative mapping analyses indicate practices within certain application contexts where methodological documentation is weak and requires improvement. Records within these patterns cannot be reproduced or interpreted quantitatively, rendering them “(methodologically) unsound”.

In the following sections, we outline the screening and selection of bibliographic records, their evaluation via the DEERS framework (outcome A), and subsequent data analyses. Quantitative measures were explored through meta-regression (outcome B), while qualitative measures were examined using multivariate statistics (multiple correspondence analysis, MCA) and machine learning (*Apriori* relational networks) to identify methodological reporting strengths and shortcomings (outcome C). Discussions pertinent to the findings are presented immediately following their introduction. Section 4 integrates these findings into a coherent synthesis, which is then used to develop a research and regulatory guidance framework (outcome D in Section 5).

### 3.1 Search, Screening, and Selection Processes

We retrieved 229 publications from the comprehensive Boolean operator search and screening processes (Figure 1). For initial piloting, including data item domain coding and refining search strings, we utilized 73 highly relevant publications previously known to the authors. After cross-referencing, this database also contributed 101 publications to the BTS3 database (“historical”). The Boolean searches yielded 39 publications in BTS1 (“endocrine”) and 89 in BTS2 (“mutagen”). This process was repeated twice, initially after 3 weeks and again after 10 months. The first repetition yielded no new outcomes, while the second added two eligible publications,^66,67^ which were not included retrospectively in our assessment.

**Figure 1:**
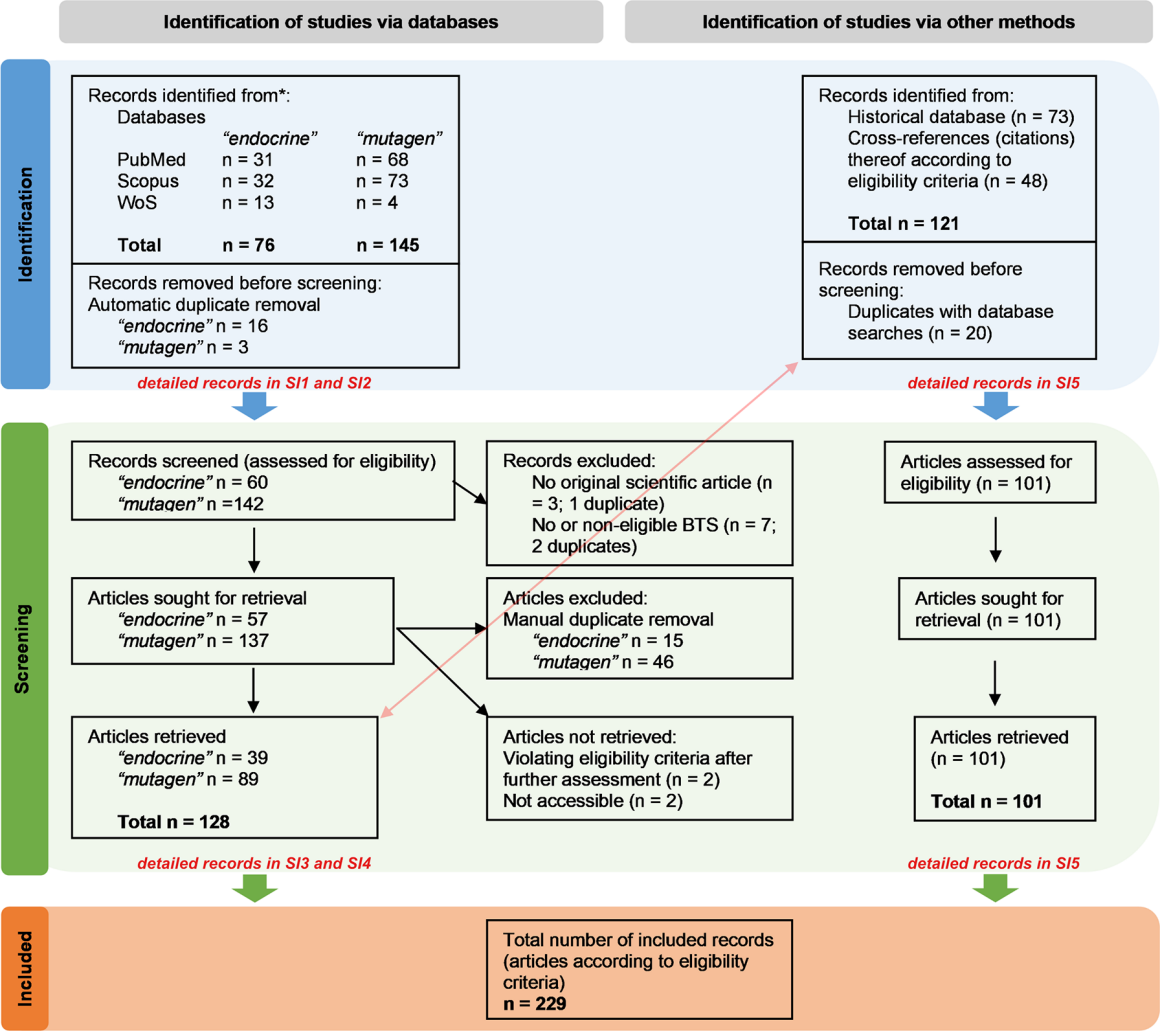
Flow diagram of the search and selection processes. The chart was designed according to PRISMA guidance.^45,46^ Detailed records are given in the supplementary information material files (SI3 to SI6). Section 3.1 of the SM outlines reasons for record exclusion according to the eligibility criteria.

The SM, Section 3.1 provides a more thorough discussion of the screening and selection processes, including the naming and reasoning of record exclusion according to the study eligibility criteria, as depicted in Figure 1. Study characteristics and citations for each bibliographical database (BTS1 to 3) are provided in SI3–SI6, and separate bibliographical database reference lists are available in the SM, Section 5.

### 3.2 Methodological Reliability Assessment and Scoring (DEERS, Outcome A)

As a result of the DEERS assessment, Figure 2A displays the hierarchical absolute scores for each article, ordered from lowest to highest. Articles are identified by their assigned ID numbers, with more detailed identifications available in SI6. Bibliographic identifiers were excluded from the main article to maintain professional courtesy and acknowledge the context of historical scientific development.

**Figure 2:**
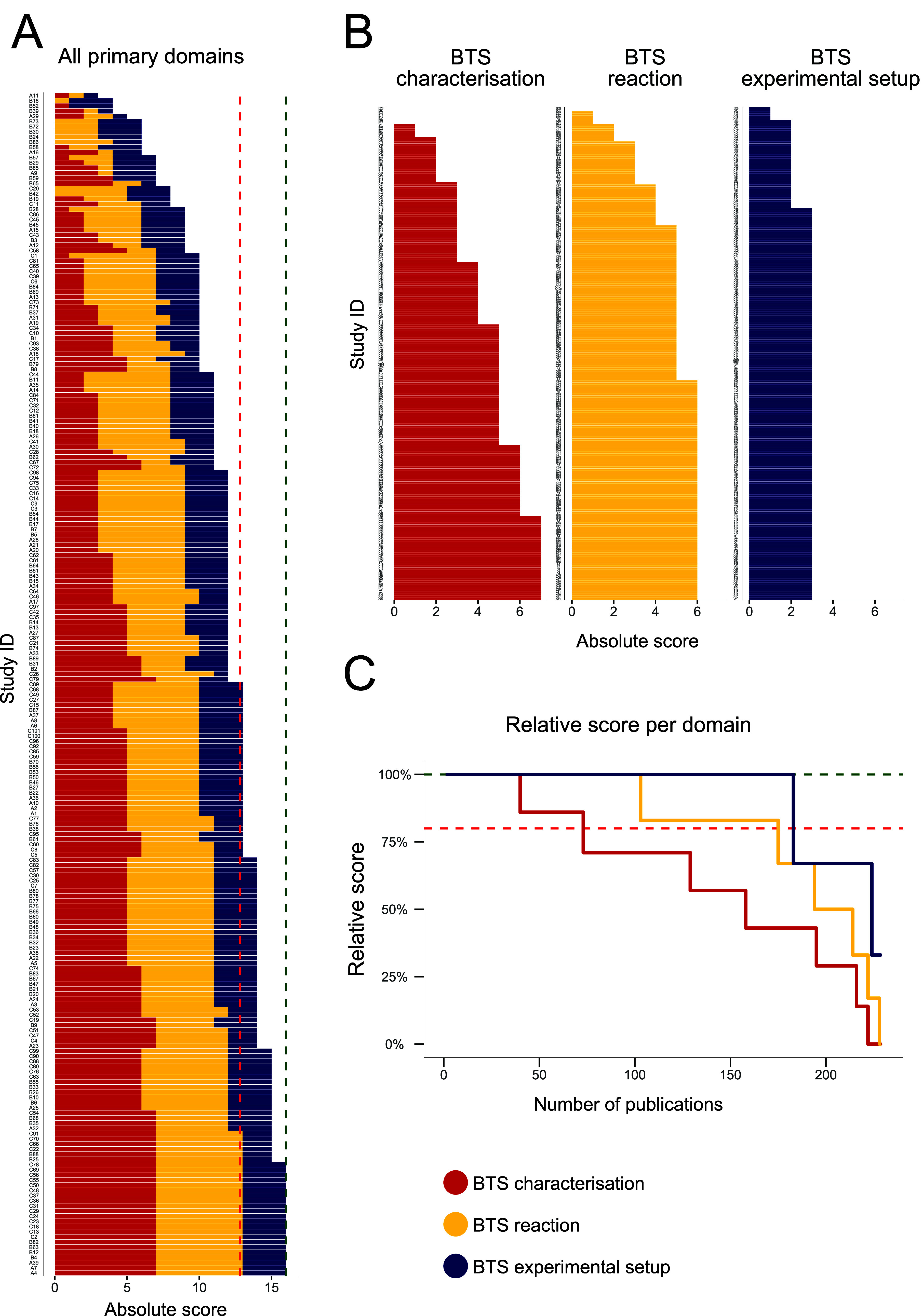
Scoring values for all *n* = 229 assessed studies via DEERS. (A) Total hierarchical absolute scores for all set studies by primary data item domains; “BTS characterization” (red, max score = 7), “BTS reaction” (yellow, max score = 6), and “BTS experimental setup” (blue, max score = 3). The red dotted line indicates a threshold of tolerable quality acceptance of 80%. Only a 100% score is considered fully reproducible and robust; see the green dotted line. (B) Separate absolute scores for the primary data item domains “BTS characterization”, “BTS reaction”, and “BTS experimental setup”. (C) Relative scores for all primary data item domains for comparison. For more details, please consult SI7. Refer to Table 1 for the respective subdomains.

Our nonweighted scoring for data item (sub)­domains revealed that only 22 publications (9.6% of total records) fully met an acceptable reproducibility standard. The scoring criteria were conceptualized so that only studies reaching 100% reporting status were considered fully reproducible, whereas an overall score of approximately 80% (translating to 13 absolute points) was considered a lower boundary. This 80% threshold was established on the rationale that studies scoring within this range could likely attain full reproducibility with minor modifications in their methodological descriptions, as detailed in Sections 4 and 5. Among the evaluated studies, a total of 115 articles attained scores of 13 points or higher. However, 114 articles (49.8% of the total evaluated studies) fell below this threshold, demonstrating a concerning level of insufficient BTS reporting quality. This indicates that half of the analyzed studies were considered not to be improvable and easily rectifiable in terms of their reproducibility.

Particularly, the “BTS characterization” primary domain exhibited the lowest scores, with only 39 studies achieving full marks and 72 surpassing the threshold (Figure 2B,C). For the ″BTS reaction”, 102 studies reached a full score, and 174 exceeded the threshold. The “BTS experimental setup” domain exhibited the highest reporting standards, with 182 studies attaining a full score and an equal number surpassing the threshold (Figure 2B,C). Within the primary domain “BTS characterization”, especially the secondary data item subdomains “strain” (69% relative score), “pooling—sex” (57%), “pooling—number of individuals” (22%), and “husbandry details” (33%) were poorly reported (see the SM, Table S5). Additionally, within the primary domain of the “BTS reaction”, the subdomain “BTS protein concentration” also scored poorly (57%). Tables S5 and S6 in the SM highlight areas where reporting standards should be, namely, improved. In contrast, subdomains within the “BTS experimental setup” primary domain were comparatively well-documented, with 87 to 95% relative scoring correspondence observed (see the SM, Table S7). Readers are referred to the SM (Section 3.2 and Tables S5–S7) for in-depth analysis and specific scoring of subdomains. Potential quality improvement measures are addressed below in Section 4.

Boxplots were generated for each primary data item domain to enhance the visualization and comparison of scoring distributions across all assessed articles. These boxplots illustrate the relative scoring, facilitating comparisons between search strategies and databases (see Figure 3 with summary statistics detailed in the SM, Table S8). The overall data set’s subthreshold mean scoring value (76%, Figure 3D and the SM, Table S8) further underscores concerns about methodology reporting. The means (74 and 73%) and medians (both 75%) of Boolean search-derived data sets (BTS1 and 2) scored below the threshold (Figures 3A,B). The overall median reached 81% due to higher methodological robustness within BTS3 (refer to Figure 3C and Figure S2 in the SM for statistics). Likewise, we encountered positive methodological robustness markers associated with the BTS3 data set within the iterative mapping analyses (outcome C, Section 3.4 below). It can be argued that BTS3 encounters some selection bias, given that it is originally based on a set of 73 publications known to the authors and deemed to be highly relevant. Accordingly, the methodological robustness might be inherently higher given their prominence. Details on potential biases are elaborated in the SM, Section 4.1.

**Figure 3:**
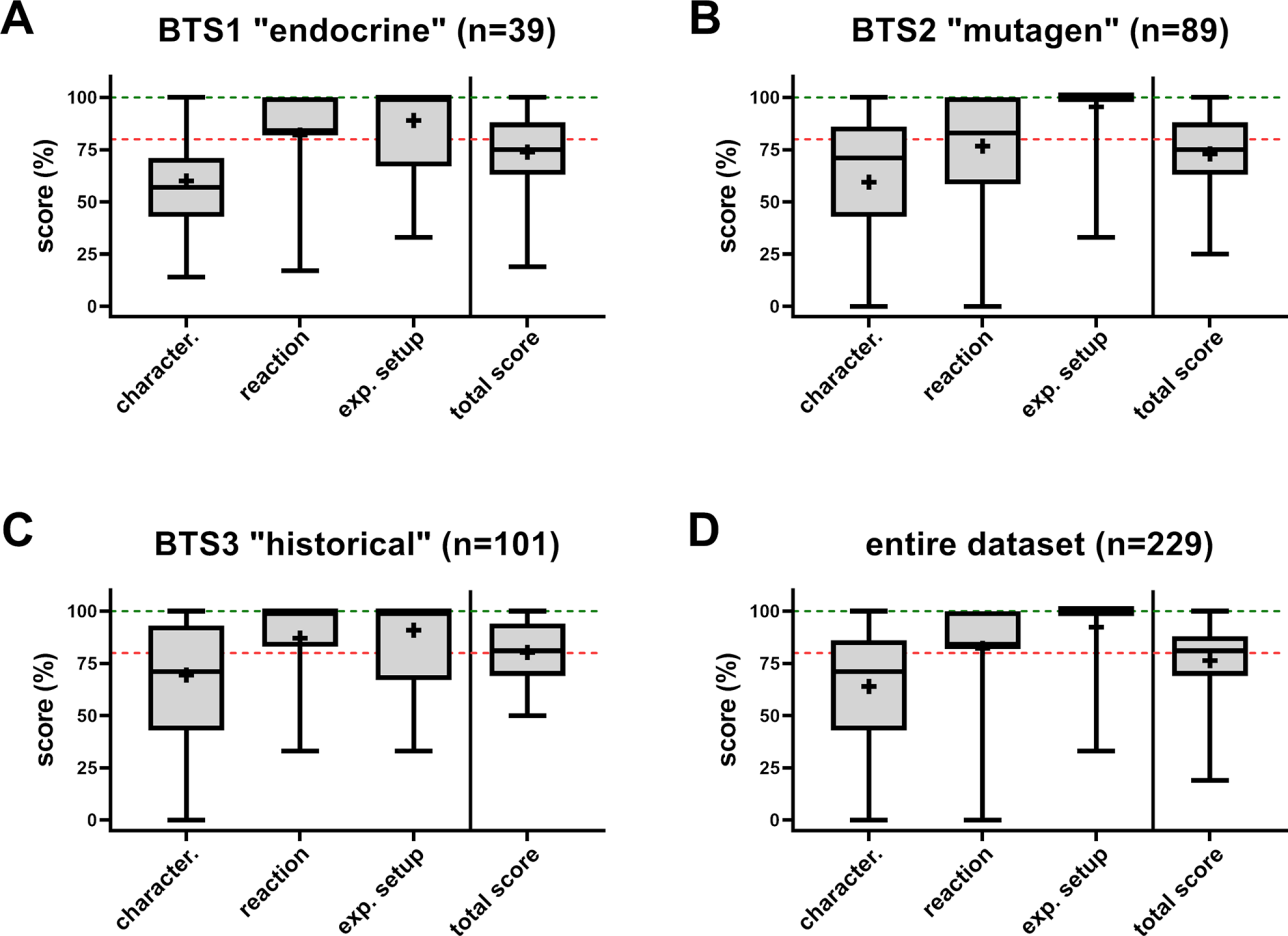
Boxplots depicting relative scoring populations of reviewed and assessed articles for all primary data item domains. Scoring distribution is given for every data set as defined by the search strategies (A, B, and C) and all data sets together (D). Whiskers indicate the upper (maximum) to lower (minimum) boundaries. Boxes indicate the 75th and 25th percentiles, and the in-between line represents the median. Crosses represent the mean scores. The upper green dotted lines indicate full data reproducibility and robustness, and the lower red dotted lines represent 80% of reporting quality thresholds. The number of included articles within every data set is given in the titles.

In summary, to date, the overall body of scientific literature has poor methodological reporting robustness. In most cases, it is not possible to reproduce or adapt for follow-up applications (e.g., *in silico* biotransformation models^68,69^ ).

### 3.3 Meta-Regression of Quantitative BTS Reaction Components (Outcome B)

In the meta-regression analyses, we focused on the numerical data item measures relating to BTS protein concentrations, primary cofactor concentrations, and BTS incubation periods. Given the hypothesized linear or exponential relationships between these data items, we investigated the potential statistical correlations between BTS protein concentration and incubation period (Figure 4A), primary cofactor concentration and incubation period (Figure 4B), and BTS protein concentration and primary cofactor concentration (Figure 4C), for the overall data set, insofar as numerical data item measures could be extracted.

**Figure 4:**
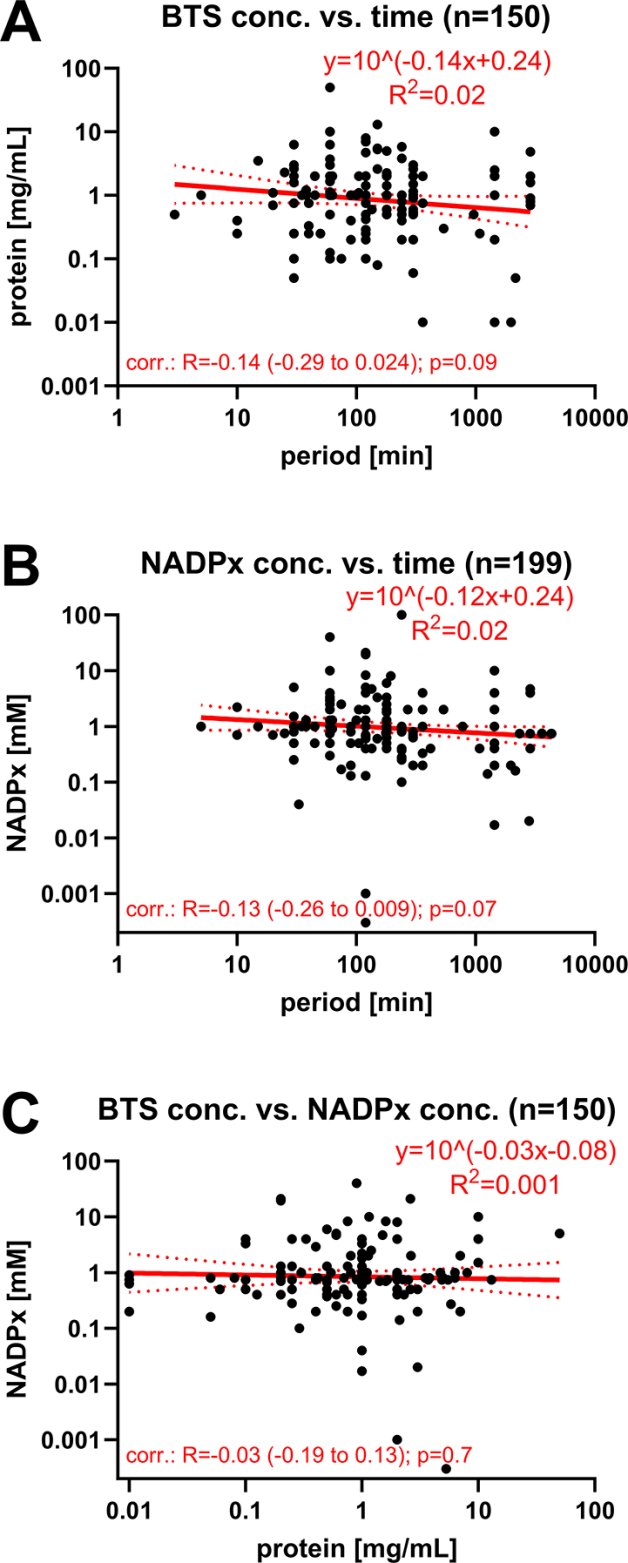
Simple linear regression fits of numerical data item measures hypothesized to show a particular mathematical relationship. Data were extracted from the overall database (SI6) and manually curated, as outlined in Section 2.7, with detailed seminal populations presented in SI8. Numerical data were log–log-transformed before fitting a simple linear regression. Equations and fits (*R* ^2^ values) are given in the respective graphs. Single measures are illustrated as dots. The seminal populations are provided within the titles. The regression fits are displayed as red lines, with 95% CIs as dotted red lines. Pearson’s correlation was computed between variables, the respective coefficients of correlation (*R*), coefficients of determination (*R* ^2^, same as for linear fit), and 95% CIs are given within the graphs. *P*-values determine the statistical significance of the correlation. The following pairs were tested: (A) BTS protein concentration vs BTS incubation period; (B) primary cofactor concentration vs BTS incubation period; (C) BTS protein concentration vs primary cofactor concentration. Additional paired or selected comparisons are given in the SM, Section 3.3, Figure S3.

Additionally, to explore the potential kinetic and dynamic impacts of cofactor regeneration systems, regression analyses were explicitly conducted for paired analyses (measures taken from the same publication record) and BTS setups that employed only NADPH, without a cofactor regeneration system (see the SM, Section 3.3, Figure S3A–C). Within the graphs, “NADPx” refers to the various forms of nicotinamide adenine dinucleotide phosphate used as reducing agents in BTS reactions.^70,71^ Thereby, NADPx is applied directly in either its reduced state (NADPH) or its oxidized form (NADP+), including a redox-cycling regeneration system. While adding NADPH directly is the more straightforward approach, integrating NADP+ with a regeneration system is both more economical and ensures a stable reducing agent concentration throughout prolonged incubation times.

Moreover, to account for phase 1 and 2 enzymatic and substrate-specific kinetic effects, subdata sets were curated by stratifying the overall data set by reaction phase (phase 1 only, and combined phase 1 and 2) and by chemical substrate (bisphenols, benzo­[*a*]­pyrene, and cyclophosphamide), insofar as numerical data item measures could be extracted for the relevant BTS parameters. Phase 2-only reactions and other substrate types yielded an insufficient number of records to allow further analyses.

Meta-regression analyses of the total extractable BTS-related numerical data item measures revealed no significant statistical relationships among the investigated quantitative measures, neither for overall (Figure 4), paired (Figure S3A–C), nor selected (without a cofactor regeneration system, Figure S3B,C) comparisons. The goodness-of-fit (*R* ^2^) values were consistently low, ranging from 0.001 to 0.2, and the regression lines were predominantly horizontal. The correlation coefficients (*R*) hovered around zero (−0.14 to 0.13), with 95% confidence intervals (CIs) spanning both positive and negative values, indicating a lack of directional correlation. The coefficients of determination (*R* ^2^) varied between 0.1 and 2%, reflecting minimal variance in one variable being explained by the variance in another. Furthermore, the *p*-values ranged from 0.07 to 0.7, indicating insufficient evidence to reject the null hypothesis of no correlation between the variables.

Similarly, analyses restricted to phase 1-only reactions (Figure S3D,E) did not yield statistically significant relationships for the investigated BTS-related variables, showing low goodness of fit (*R* ^2^ = 0.007 to 0.01) and no significant directional correlation (*p* = 0.22 to 0.46). A comparable absence of statistically significant relationships, poor model fits, horizontal regression lines, and lack of directional trends was also observed for the bisphenol-, benzo­[*a*]­pyrene-, and cyclophosphamide-stratified subdata sets (Figure S3K–R). The only exceptions were the relationships between BTS protein concentration and incubation time for bisphenol substrates (Figure S3, panel J) and between NADPx cofactor concentration and incubation time for the cyclophosphamide subset (Figure S3, panel Q). Both showed statistically significant directional correlations (*p* = 0.003 and 0.03, respectively) with acceptable goodness of fit (*R* ^2^ = 0.60 and 0.51, respectively). However, the regression for bisphenols was opposite to the hypothesized direction (increasing BTS concentrations with longer incubation times instead of decreasing), while the cyclophosphamide fit was limited by a small sample size (*n* = 9).

In contrast to the previously identified trends, meta-regression analyses restricted to combined phase 1 and 2 reactions revealed statistically significant correlations for incubation period, BTS protein concentration, and NADPx cofactor concentration-related variables, with the expected hypothesized directionality and adequate sample sizes (*n* = 38 and 36, respectively) (Figure S3G–I). However, despite these significant correlations (*p* = 0.006 to 0.04), the data exhibited considerable scatter. Moreover, directionality and significant correlation were largely driven by outlier data points (Figure S3G–I), as reflected by the low goodness-of-fit values (*R* ^2^ = 0.11 and 0.15, respectively).

We conclude that overall, the meta-regression analyses indicate limitations in demonstrating robust correlations between the key quantitative components of BTS reactions. Nevertheless, some trends appear to be emerging when parametrization is examined at higher resolution, particularly with respect to the applied NADPx concentrations across the range of BTS incubation periods (Figure S3H,Q).

Thus, it is uncertain whether proper assessments of concentration–response and reaction condition components of BTS-induced biotransformation processes have been thoroughly conducted throughout the extensive literature. Instead, component selection seems influenced by historical protocols^31–33^ and has been continuously pursued, or the selection happened in a rather *ad hoc* manner, as demonstrated by numerical BTS parameters ranging over several orders of magnitude without correlative trajectories (Figure 4 and Figure S3A–F). The inclusion of all reaction parameters (substrate concentration, BTS protein concentration, and cofactor concentration at different levels) is unrealistic to test and assess, if not the prime target of the investigation, due to time and cost limitations. To our knowledge, only one study^72^ comprehensively set these parameters using a “Doehlert” experimental design matrix. This indicates the need for more detailed investigations into the BTS reaction dynamics to set appropriate standards.

### 3.4 Iterative Mapping Analyses of Qualitative Data Items (Outcome C)

After further coding simplification (detailed in the SM, Table S4, and SI9), qualitative data item measures were made machine-readable and forwarded to data mapping analyses. As such, the data was subjected to descriptive statistics (represented as histograms, Euler plots, and Upset plots), MCA, and *Apriori* algorithms (for data association and relational networks).

#### 3.4.1 Descriptive Statistics of Qualitative Data Item Subdomains

Descriptive statistics of qualitative data item subdomains (more details and illustrations in the SM, Section 3.4) revealed evolving dynamic trends over time, including shifts in publication focus (see the SM, Figure S4), diversification of study end points (SM, Figure S6), and changes in the species used for BTS (SM, Figures S9 and S10). A notable coincidence was observed between external sourcing of BTS (SM, Figure S8) and a decline in reporting accuracy for certain subdomains (“strain”, “BTS pooling”, and “BTS induction”; SM, Figures S11, S12, and S14).

Another pattern identified within the descriptive statistics analyses relates to the primary cofactor (“NADPx”) regeneration systems.^70,71^ Noteworthily, oxidized NADP+ is utilized alongside glucose-6-phosphate (“G6P”) or isocitrate (“iso.”) for redox-cycling regeneration systems. Our analysis primarily focused on identifying whether these regeneration systems were mentioned. Detailed components of these systems, particularly dehydrogenases (“dh”), were not exhaustively scored to avoid overcomplicating the assessment. However, it is critical to note that for a reproducible system, both the presence and concentrations of applied dehydrogenases should be reported. Of 177 studies using cofactor regeneration systems, only 35 studies provided comprehensive details on the essential dehydrogenase components (see the SM, Figures S16–S18).

We also encountered inconsistencies in the literature regarding the use of cofactors for phase 1 or 2 biotransformation studies. Several studies allegedly examining phase 2 reactions predominantly utilized phase 1 inducing systems (NADPH or NADP+ with regeneration systems). Conversely, some research focusing on phase 1 metabolism incorporated phase 2 specific cofactors (e.g., GSH) into the final BTS reaction mixture. Furthermore, there were instances in which studies claimed to explore specific phase 2 reactions that are impractical to assess in mitochondrial fractions (microsomes). With respect to the intricacies of cofactor utilization, the reader is directed to relevant literature, e.g., refs 19,73,74 . In conclusion, there is a pressing need for substantial enhancements in reporting primary cofactors and their regeneration systems to meet the adequate quality for BTS applications.

#### 3.4.2 Multiple Correspondence Analyses (MCA) of Qualitative Data Item Subdomains

Qualitative data measures underwent MCA across various subdomains to discern patterns among categorical interdependent (active) and independent (supplementary) variables. MCA facilitates the reduction of data dimensionality analogous to principal component analysis (PCA), yet it is distinctively suited for qualitative categorical variables. Notably, the variables “publication year”, “publication group” (data sets BTS1–BTS3), and “total scores” were treated as supplementary variables, thus exerting no influence on the outcome of the analysis. The two principal dimensions (Dim.1, Dim.2) identified by MCA explain most of the variance (21.7% total, 13.3 and 8.4%, respectively, e.g., Figure 6) within categorical experimental parameters among the entire data set, with publication records similar in experimental parameter selection clustering together.

Notably, it has been described that MCA underestimates the apprehended variance, as the underlying computation introduces additional inertia.^75,76^ As a rule of thumb, the explained variance computed via MCA can be multiplied by a factor of 2 for comparison with familiar PCA values. Thus, the explained variance computed here is reasonable, especially for a multidimensional data set. Moreover, the variance is of secondary relevance; the emerging patterns were considered to be of greater interest.

Superimposing the MCA computations with the supplementary variable “total score” revealed that publications comparable in methodological parameter selection are also similar in scored robustness (Figure 5A). Furthermore, we observed that the line of unity roughly divides the data set into segments of acceptable and nonacceptable methodological reporting rigor, when the 80% threshold criterion (see Section 3.2) is applied (equivalent to 12.8 or 13 absolute points, Figure 5B). Therefore, variable patterns with centroids to the right of the line of unity, not or barely intersected by their 90% confidence interval (CI) ellipses, are considered robust, and *vice versa*, variable patterns with identical centroid and CI ellipses characteristics to the left of the line of unity as unsound. Especially patterns clustering in the lower right quadrant can be, in majority, identified as robust, whereas patterns in the upper left quadrant tend to be unsound. This delineation via the line of unity was further utilized to identify experimental parameters linked to robustness patterns among the qualitative data item subdomains. Here, we focus on subdomains with distinct relational patterns, with additional data in Section 3.5 of the SM. To enhance readability and graphical identification, not all CI ellipses were plotted; only ellipses of the discussed categories were plotted. For complete illustrations, please consult the SM, Section 3.5, Figures S21−S24.

**Figure 5:**
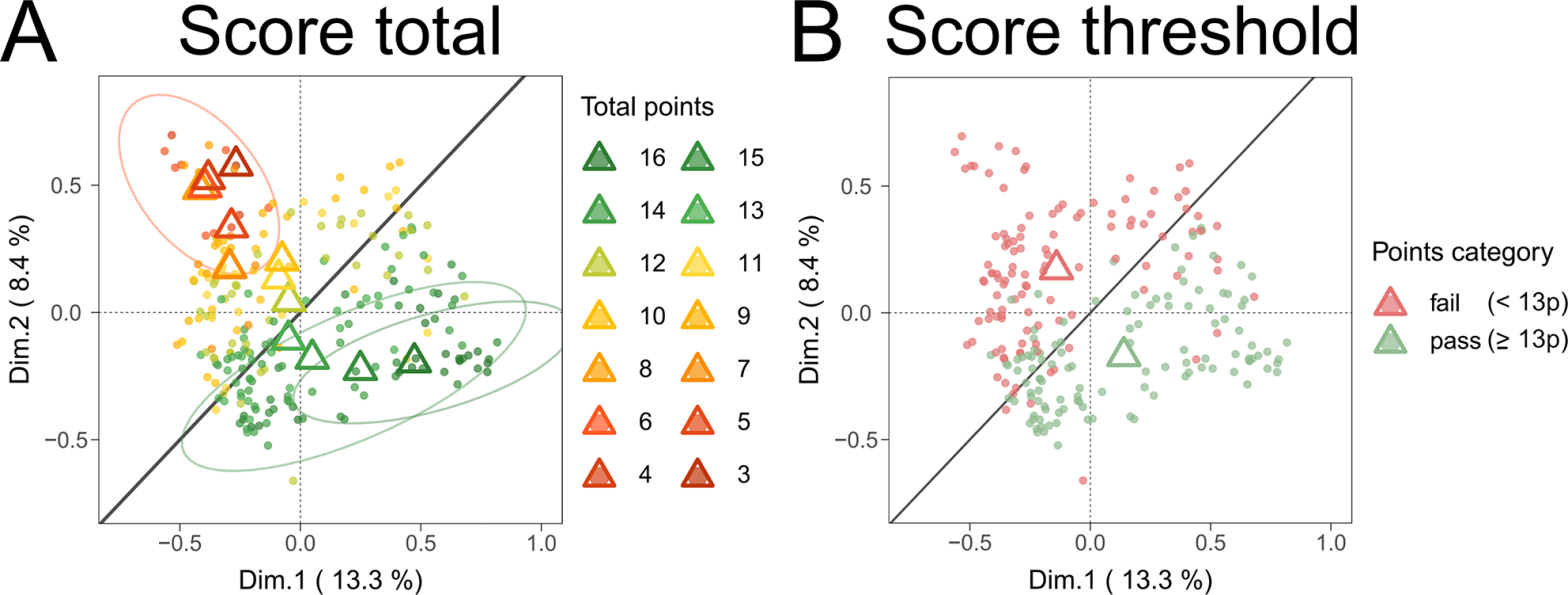
Multiple correspondence analysis (MCA) plots showing the clustering of *n* = 229 publications regarding the total attributed score (A) or divided by the 80% threshold (B). Centroids (respectively colored triangles) mark the mean individual dimensional coordinates per variable. Ellipses (colored) represent normal probability contours at a 0.9 confidence level. The line of unity is given in dark gray.

MCA identified patterns of methodological reporting robustness and unsoundness within qualitative subdomain measures, hinting at the shortcomings in the methodological reporting of external BTS applications while also indicating possible solutions.

Robustness markers were identified in the “BTS characterization” primary domain, when examining the subdomains “BTS origin” (producer and system origin), “species”, “pooling” (sex), “husbandry”, and “BTS induction” (Figure 6). For “BTS origin”, internally produced BTS showed more robust trends compared to externally purchased ones (Figure 6B), and *in vitro*-derived BTS (e.g., derived from hepatocyte cultures) exhibited higher reporting soundness (Figure 6A). In the “species” subdomain, fish and human-derived BTS systems showed greater robustness than rat-derived and undefined (“nd”) systems (Figure 6C). Regarding “pooling”, combinations of “female and male” or “female only” systems were more robust than “male only” or undefined (“nd”) systems (Figure 6D). Records reporting husbandry and enzymatic activity related to biotransformation displayed a higher robustness (Figure 6E). Finally, noninduced BTS, likely correlating with human and fish-derived systems, had higher robustness than induced systems (Figure 6F). Especially when compared to classical BNF/PB and Aroclor-induced BTS (“PCB-PAH”; note that Aroclors, other PCBs, and methylcholanthrene (PAH) were summarized in this category for MCA analysis to meet computation criteria, more details are given in the SM, Section 2.12, Table S4, and SI9).

**Figure 6:**
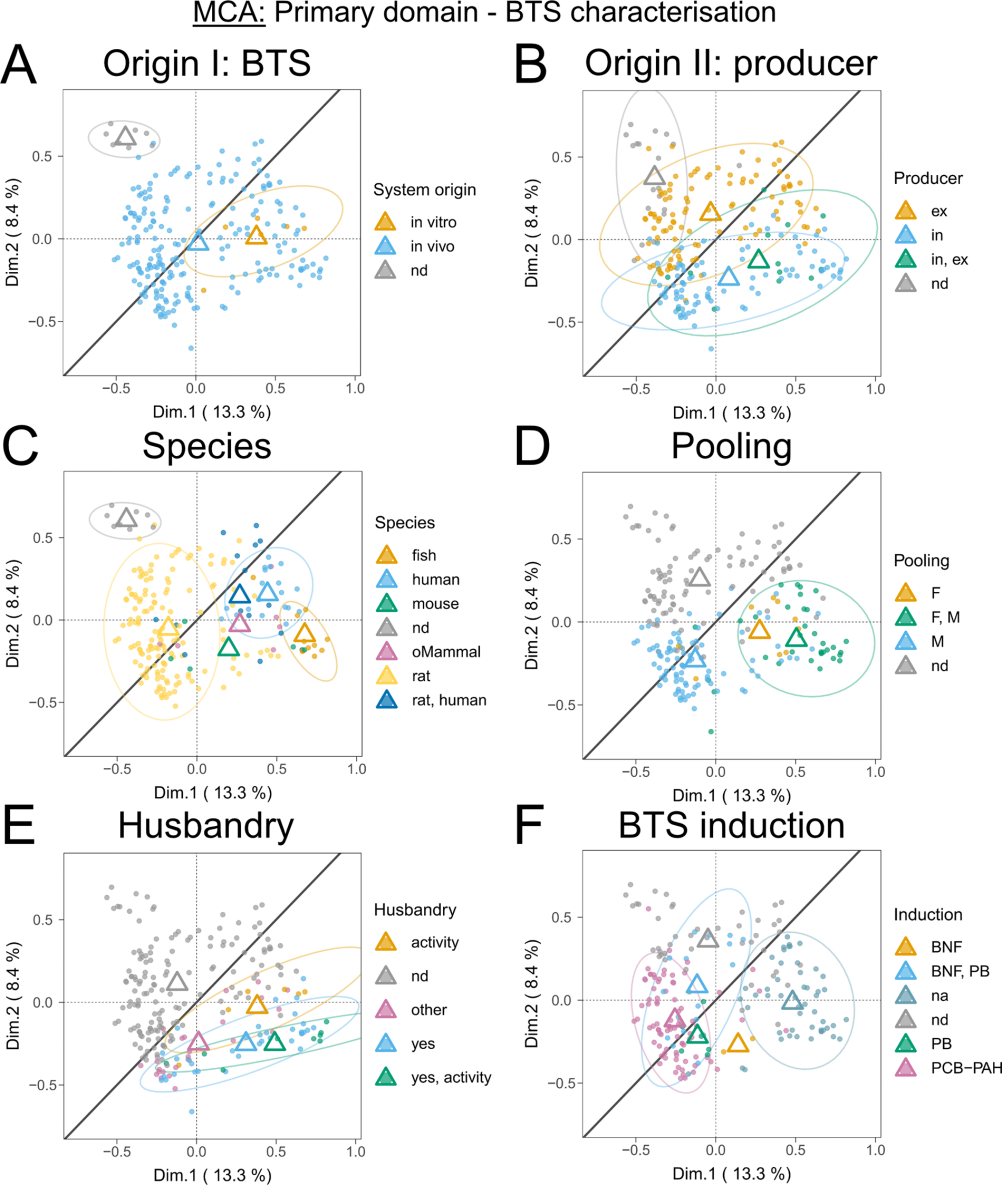
Multiple correspondence analysis (MCA) plots showing the clustering of *n* = 229 publications by the primary data item domain “BTS characterization”. Depicted are clusters of the inquired subdomains “BTS origin” (BTS system origin, panel A; producer, panel B), “species” (C), “pooling” (D), “husbandry” (E), and “BTS induction” (F). Centroids (respectively colored triangles) mark the mean individual dimensional coordinates per category. Ellipses (colored) represent normal probability contours at a 0.9 confidence level. The line of unity is given in dark gray. Abbreviations: nd—not defined; na—not applicable; ex—external; in—internal: oMammal—other Mammals; F—female; M—male; BNF—beta-naphthoflavone; PB—phenobarbital; PCB-PAH—polychlorinated biphenyls and polycyclic aromatic hydrocarbons (note that Aroclors, other PCBs, and methylcholanthrene (PAH) were summarized in this category for MCA to meet computation criteria, more details are given in the SM, Section 2.12, Table S4, and SI9).

We also encountered robustness markers in the primary reliability domains “BTS reaction components”, subdomains “solvent” (Figure 7A), with studies employing organic solvents, and “BTS experimental setup”, subdomain “BTS-related control” (Figure 7B), for records featuring the categories “w/o cofactors” and “inactivated BTS”. In general terms, for studies utilizing two BTS-related controls, all centroids were located right to the line of unity, whereas only employing a single control was located to the left. The subdomain “cofactor class” depicted a robust pattern for relative observations investigating only phase 2 metabolism (Figure S22B). However, an ellipse could not be computed due to the low number of relative observations (*n* = 3).

**Figure 7:**
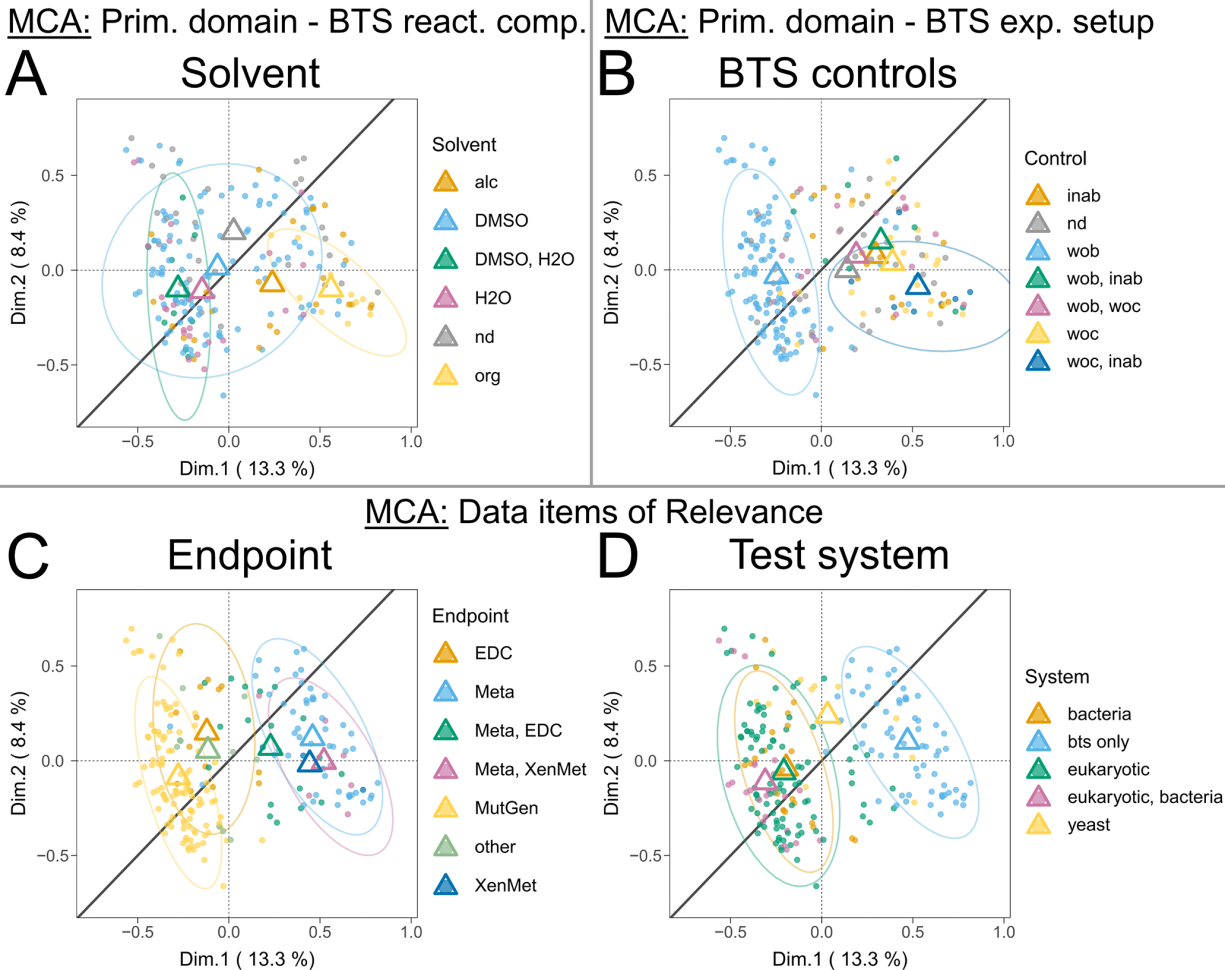
Multiple correspondence analysis (MCA) plots showing the clustering of *n* = 229 publications by the primary data item domains “BTS reaction components”, “BTS experimental setup”, and data item domains of relevance. Depicted are clusters of the inquired subdomains “solvent” (A), “BTS-related controls” (B), “endpoint” (C), and “test system” (D). Centroids (respectively colored triangles) mark the mean individual dimensional coordinates per category. Ellipses (colored) represent normal probability contours at a 0.9 confidence level. The line of unity is given in dark gray. Abbreviations: nd—not defined; alc—alcohol-based solvent; DMSO—dimethyl sulfoxide-based solvent; H_2_O—water-based solvent; org—organic solvent; inab—inactivated BTS; wob—without BTS; woc—without cofactors; EDC—endocrine disruption; Meta—metabolites; MutGen—mutagenicity and genotoxicity; XenMet—xenobiotic metabolism functionality.

Robustness markers were also observed in the “endpoint” and “test system” relevance domains (Figure 7C,D). Studies focusing on “metabolites” (identification) and “xenobiotic metabolism” (enzymatic activity) end points depicted higher robustness (Figure 7C). BTS-only test systems generally exhibited higher methodological reporting robustness (Figure 7D), particularly when compared to test systems that employed eukaryotes and bacteria.

However, markers of unsoundness were less evident, with mainly nondefined (“nd”) data item measures per subdomain (e.g., Figure 6A–C) clustering in the upper left quadrant. Besides that, negative trends are identified for categories with centroids left of the line of unity but crossing ellipses. Specifically, these include rat-derived BTS (Figure 6C), BNF/PB and PCB or PAH-induced BTS (Figure 6F), DMSO + H_2_O solvent-utilizing test systems (Figure 7A), setups using only the “w/o BTS” control (Figure 7B), and studies focusing on mutagenicity, genotoxicity, and endocrine disruption end points (Figure 7C), all depicting negative robustness tendencies.

For other qualitative data item subdomains, the overall methodological quality of reporting patterns has remained consistent across various scientific fields over time, as no or only a minor discernible robustness pattern direction could be established. Other patterns also seemed to be less critical for our analyses. Additionally, the subdomains “strain” and “buffer system” are further discussed in the SM, Section 3.5.

The MCA’s emerging patterns are instrumental in pinpointing specific areas of methodological reporting soundness and shortcomings and provide a platform for developing procedures to address and potentially rectify these issues (as further discussed in Section 4). In summary, the subdomains “BTS origin”, “BTS induction”, “BTS controls”, “pooling”, “husbandry”, “solvent”, and “species” showed significant divergence throughout positive robustness markers (Figures 6 and 7). Although less evident, we also identified positive robustness trajectories for the subdomains “test system”, “endpoint”, “cofactor (class)”, and “strain”. Internally produced, *in vitro*, fish, or human-derived, and nonchemically induced BTS yielded stronger robustness patterns.

#### 3.4.3 Data Association Rule Mining and Relational Networks via *Apriori* Algorithms

Association data rule mining, utilizing *Apriori* algorithms, was implemented to discern relational networks among qualitative data item subdomains. These algorithms identify sets of qualitative data item subdomain measures that frequently coincide and establish associative connections (rules) between them. The “support” value quantifies their co-occurrence, reflecting the frequency of interactions between measures. Another key metric, “lift”, indicates the strength of item associations. Lift values above 1 signify a positive association, indicating co-occurrence beyond random expectation. Absolute scores “pass” (≥13 points) and “fail” (<13 points), as derived from the 80% relative score criterion threshold, were included in the analyses as parameters to force network anchoring and facilitate interpretation, as otherwise, the networks would hardly be interpretable to the human inspector.

Figure 8 showcases a relational data network constrained to the 50 most prominent rules. Anchored by scoring robustness, the network bifurcates into two distinct clusters, one representing high robustness (right-hand side) and the other representing poor reporting quality (left-hand side), with both converging at the central association of the “*in vivo*-derived BTS system origin”.

**Figure 8:**
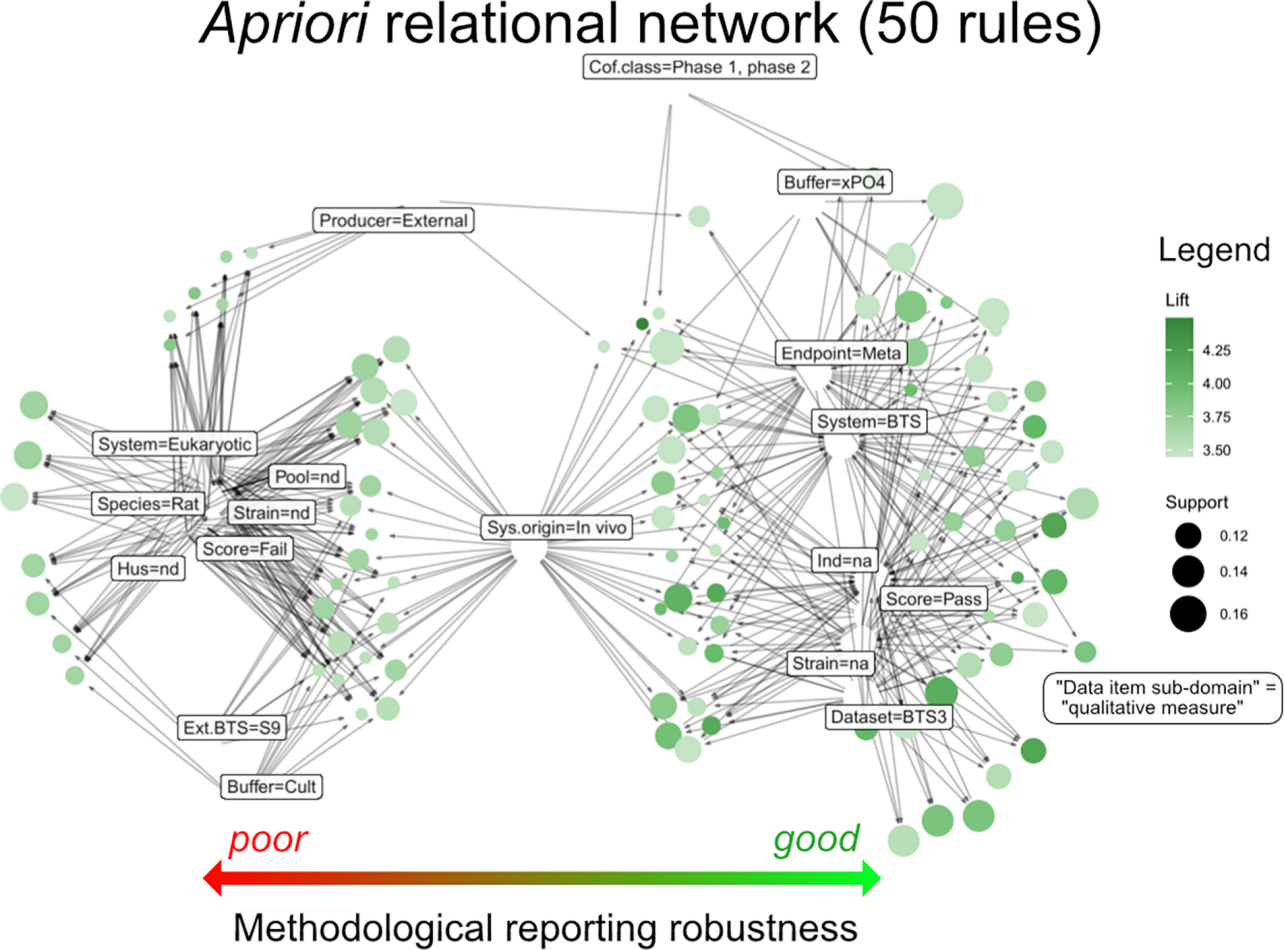
Relational data network derived from all qualitative subdomains included in the simplified, machine-readable coding book (the SM, Table S4 and SI9). Patterns were emphasized by anchoring; relational networks to the left are related to poor methodological reporting reliability, while clusters to the right are robust. The network comprises records from *n* = 229 publications. Fifty rules (association connections) are depicted. Rules are shown as dots, with the size illustrating the support and the color representing the lift of the association. Arrows indicate the direction of the rules, as they are associated with frequent data item measures (squares). The data item measures are located within the voids but were moved to increase readability. Rule mining was conducted at a support threshold of 10% and a confidence threshold of 80%. Interactive plots for 100 to 5000 rules are given in SI10. Abbreviations: nd—not defined; Cult—cultivation medium; Cof.class—cofactor class; xPO4—generic phosphate buffer; Meta—metabolites, na—not applied; BTS3—data set BTS3/historical.

In the robust pattern, methodological reporting strength correlates with the exclusive use of “BTS (-only)” systems (excluding other *in vitro* systems), assessing “metabolites” as end points, absence of chemical induction, and nonapplicability of rodent strains—suggesting nonrodent BTS origins. This pattern is also loosely associated with using phosphate buffers and examining both phase 1 and 2 metabolism. Notably, the “BTS3/historical” database links to higher robustness, a fact that is pursued in the bias discussion (SM, Section 4.1).

Conversely, the poor-quality pattern aligns with undefined measures (“nd”). Interestingly, other defined measures cluster in direct proximity, such as rat-derived BTS and test systems incorporating “eukaryotic” cells in conjunction with the BTS. Further associations include “S9 BTS”, externally sourced BTS, and systems utilizing cellular culture medium as a buffer.

The supplementary material folder SI10 includes interactive networks based on 100 to 5000 rules. In the 100-rule network, robustness patterns mirror those in the 50-rule network (Figure 8). The 200-rule network reveals additional negative subdomain to data item measure associations, such as “BTS-related control” to “w/o BTS” (without BTS) and “cofactor class” to “phase 1”, near lower scores. At 500 rules, new clusters emerge (“dataset” to “BTS2” (BTS2/mutagen), “endpoint” to “MutGen” (mutagenicity and genotoxicity), “solvent” to “DMSO”, and “field” to “Tox” (classic toxicology)), albeit closer to lower methodological reporting robustness. Beyond 500 rules, score anchoring loses relevance and the complexity renders the patterns uninterpretable by human analysis.

In summary, robustness correlated with the exclusive use of “BTS-only” test systems, assessing “metabolites” as end points, absence of chemical induction, and nonrodent BTS origins. Poor reporting patterns were associated with undefined measures (“nd”), the use of rat-derived BTS, studies focusing on the end points mutagenicity and genotoxicity, records employing culture medium as a buffer system, and studies utilizing eukaryotic *in vitro* systems in combination with BTS.

#### 3.4.4 Follow-up, Confirmatory Analyses

With the help of the explorative mapping analyses, we discerned methodological reporting robustness patterns and markers, which defined directions of scientific rigor and soundness (positive/negative). Table S9 in the SM, Section 3.7 summarizes robustness markers for identified qualitative data item subdomains and their respective measures. For subdomains that illustrated distinctive positive or negative robustness patterns in MCA and *Apriori* assessment frameworks simultaneously, we followed up with confirmatory analyses^65^ and effect size measures of the respective seminal scoring populations to consolidate the mapping results.

In eight of the nine subdomains, significant scoring population variances emerged upon examining specific measure combinations (Figure 9). For the “BTS system” subdomain, studies employing “only BTS” in a buffer system demonstrated greater methodological reporting robustness than those using “eukaryotic” cells in conjunction with BTS or other test systems (Figure 9A). For studies investigating the end points “xenobiotic metabolism” and “metabolites”, robustness was notably higher compared to “mutagenicity and genotoxicity” and “endocrine disruption” studies (Figure 9B). “Internally” produced BTS outperformed “externally” acquired BTS (Figure 9C). Fish- and human-derived BTS were superior in robustness to those from other organisms, particularly rats (Figure 9D), with fish-derived BTS scoring the highest in mean and median absolute scores. *In vitro*-derived BTS (e.g., from hepatocyte culture) ranked higher than classically *in vivo*-derived BTS systems (Figure 9E). Noninduced BTS showed a generally higher absolute score than chemically induced BTS (Figure 9F). Records utilizing phosphate buffer outperformed those with culture media (Figure 9G). Lastly, employing two or more BTS-related control types (without BTS, without cofactors, or with inactivated BTS) was more advantageous than using a single BTS-related control type (Figure 9H). No significant difference emerged in studies focusing solely on phase 2 metabolism compared to phase 1 metabolism (the SM, Section 3.7, Figure S25). However, the lack of statistical significance in the latter comparison is likely due to the low power of the nonparametric test and the small sample size in one of the comparator groups (phase 2 metabolism), despite the effect measures demonstrating a clear divergence.

**Figure 9:**
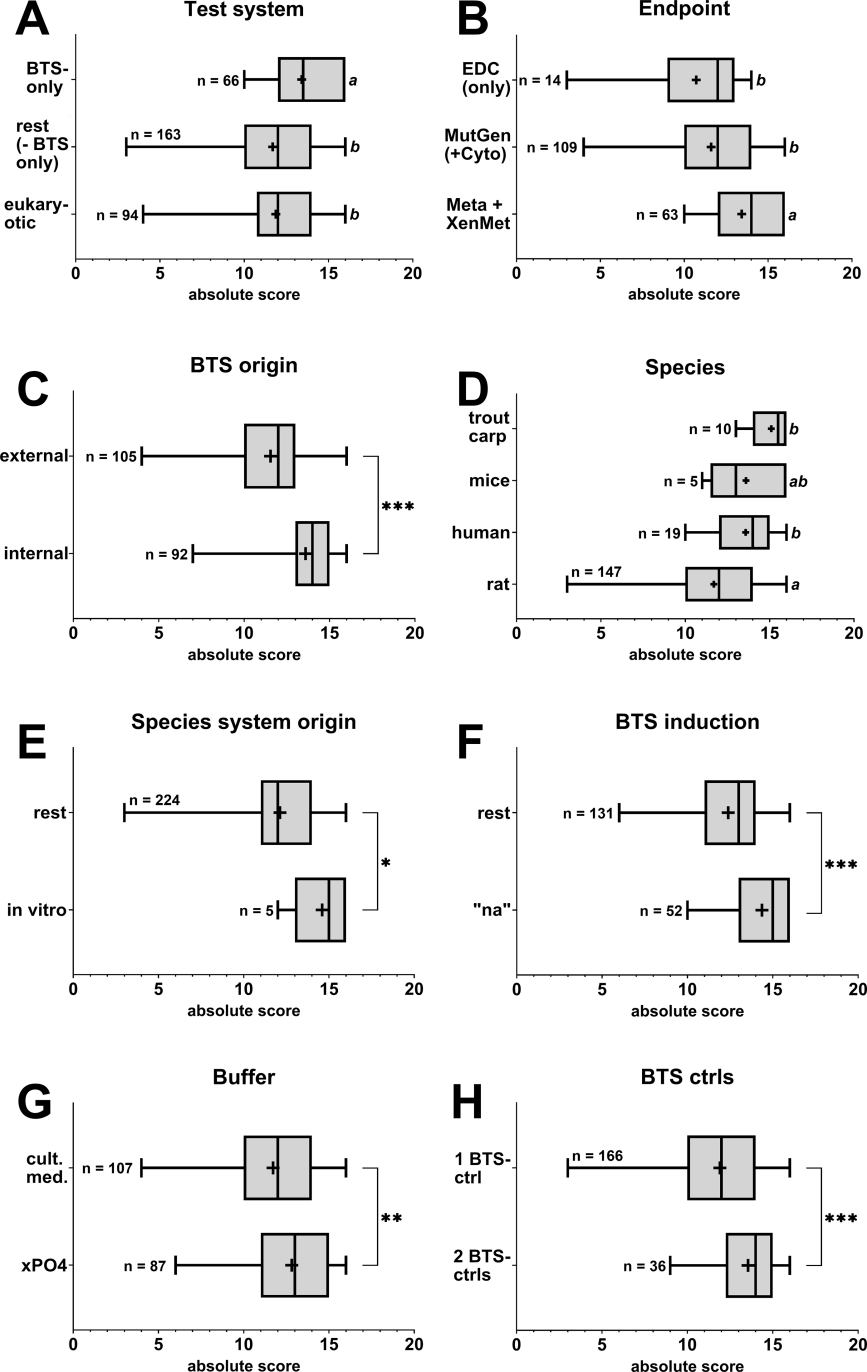
Boxplots depicting absolute scoring populations of reviewed and assessed articles for respectively curated subdata sets per qualitative data item subdomain. Whiskers indicate the populations’ upper (max.) to lower (min.) boundaries. Boxes indicate the 75th and 25th percentiles, and the in-between line represents the median. Crosses represent mean population values. Statistical analyses of variance between data sets were conducted via two-sided Mann–Whitney *U* tests (pairwise comparison, alpha level = 0.05) or Kruskal–Wallis tests, followed by Dunn’s post hoc test (multiple comparisons, alpha level = 0.05). Asterisks indicate statistically differing significance between means of respective data sets for pairwise comparisons (*p < 0.05, **p < 0.01, ***p < 0.001). For multiple comparisons, varying letters indicate statistical significance (*p* < 0.05). The number of included records per seminal population analysis is given in the graphs. More details are provided in SI11. Abbreviations: EDC—endocrine disruption; MutGen—mutagenicity and genotoxicity; Cyto—cytotoxicity; Meta—metabolites; XenMet—xenobiotic metabolism functionality; “na”—not applied; xPO4—generic phosphate buffer; cult. med.—culture medium; ctrl—control.

A previous methodological harmonization for *in vitro* hepatic clearance studies also identified the variables “concentration”, “species”, and “culture medium” as most influential in a random forest regression analysis.^77^ Overall, the results from the MCA, *Apriori*, and confirmatory analyses corroborate each other.

In conclusion, follow-up analyses on scoring population effect sizes confirmed the iterative mapping observations, showing statistically significant differences across subdomains, which reinforces the robustness markers identified in earlier analyses. Altogether, these findings are synthesized in the following sections to build a foundation for the subsequent guidance framework.

## 4 Synthesis of Results and Discussion

From the DEERS scoring assessment (outcome A), we deduced that the overall body of literature has a poor methodological reporting status regarding the application of BTS. Additionally, meta-regression analyses (outcome B) revealed a significant lack of correlations between key components of the BTS reaction. The iterative mapping analyses identified clear patterns of methodological reporting robustness (outcome C) and unsoundness, which we substantiated in subsequent confirmatory analyses of effect sizes. In the following sections, we synthesize these results and propose practical ways to enhance BTS reporting. The ensuing suggestions are structured around primary data item domains, offering targeted recommendations for improving reporting practices.

### 4.1 “BTS Experimental Setup” Is Overall Adequate

The primary domain, “BTS experimental setup”, was noted for its high level of methodological reporting rigor (Figures 2 and 3). Within this primary domain, the subdomain “BTS-related controls” emerged as a key area for potential improvement, scoring a mean relative score of 87% compared to 95% for the other two subdomains (the SM, Table S7). Positive robustness patterns were associated with the use of at least two BTS-related controls (Figures 7B and 9H). However, this did not lead to different scoring, as solely one BTS control was considered sufficient for positive evaluation and, hence, had no influence on the absolute score per record.

Within the analyzed body of literature, we identified several types of BTS-related controls, which are being employed to differentiate from fully active BTS (BTS plus respectively used cofactors). Table S11 in the SM provides detailed descriptions of various BTS-related control setups that are employed alongside prokaryotic or eukaryotic test systems. For data parametrization and coding procedures, we classified BTS-related controls into three categories, as follows: “w/o BTS” (per se the plain *in vitro* assay—without BTS and without cofactors), “w/o cofactors” (with BTS but lacking cofactors), and “inactivated BTS” (heat or chemically treated; cofactors are optional in relation to the test setup, see Table S11). Although this categorization does not capture the full complexity of BTS-related controls, which is influenced by the parallel *in vitro* systems used, post-BTS incubation procedures, and the research questions, it was adequate for our analysis and interpretation, serving as a basis for further recommendations.

Each control type plays a crucial role in evaluating different aspects that might influence the BTS-driven biotransformation process. We address the critical aspects of BTS controls for setups that incorporate externally added BTS into pro- or eukaryotic test systems, which are also applicable to assays that use cell lysates or extracts in conjunction with the BTS. Notably, standalone BTS applications are not a primary subject of this review and may require specific control strategies, which are beyond the scope of this article. The “w/o BTS” control is essential for distinguishing between systems with and without induced biotransformation.^78–80^ As such, it is used to determine whether the substance undergoes biotransformation and whether this process alters its bioactivity (within the practicable margins of the test setup). The “w/o cofactor” control accounts for the impact of self-sustained biotransformation potential within the microsomes or S9 fractions themselves, due to the low inherent quantities of cofactors remaining as residues from homogenization.^81^ This might be specifically important for studies utilizing short BTS incubation times (e.g., 10 min) or focusing on specific phase 1 or 2 enzymatic activities. Especially for S9 fractions, other biotransformation reactions may occur simultaneously, in addition to those specifically investigated by adding respective phase 1 or 2 cofactors. Lastly, the “inactivated BTS” control is crucial for assessing the impact of BTS addition on the bioavailability of chemicals within the test system. BTS addition can significantly alter the protein content and, consequently, the distribution of hydrophobic/lipophilic compounds.^82,83^ The additional protein may act as a sink compartment for hydrophobic/lipophilic compounds and impact the actual free concentration of the chemical under study.^84^ This is especially crucial in studies using complex culture media as buffer systems, such as those employing BTS alongside eukaryotic *in vitro* systems, for example, reporter-gene assays. Additional BTS control considerations are especially required when developing and troubleshooting novel methods, which are further addressed in the SM, Table S11. Overall, it ultimately rests in the experimenter’s judgment to carefully select the optimal setup of necessary controls, which needs to be clearly described in the methodology.

Given the results of our exploratory analyses and the preceding discussion, we advocate for the inclusion of at least two BTS-related controls. Experimenters should consistently include both “w/o BTS” (per se, the plain *in vitro* assay) and “inactivated BTS” (without cofactors) controls, especially in studies employing BTS alongside pro- or eukaryotic test systems, to account for total biotransformation impact and bioavailability issues.

### 4.2 Minor Improvements Can Have a Significant Impact, “BTS reaction”

In our analyses of the “BTS reaction” primary domain, it was observed that all subdomains, except for “BTS protein concentration”, were somewhat satisfactorily described, with relative scores ranging from 81 to 97% (SM, Table S6). However, the “BTS protein concentration” subdomain significantly lagged, achieving only a 57% relative score. Accurately specifying the BTS protein concentration in mg/mL is vital for several reasons. First, it enables the normalization of results across various study end points, enhancing the comparability of different studies.^21,85,86^ Second, elevated concentrations of BTS can induce cytotoxic effects, which are particularly problematic in studies involving prokaryotic or eukaryotic test systems.^87,88^ Also, an improved definition of cofactor-related measures is needed overall, for which detailed insights have already been provided above in Section 3.4.1.

A common shortfall in many studies is reporting the BTS concentration as a percentage of the final BTS reaction mixture (% v/v) without specifying the initial concentration. This approach is likely rooted in historical protocols by Ames et al.*,* ^31–33^ which introduced the Ames mutagenicity test, a significant catalyst of BTS utilization within toxicology. Ames et al.’s initial protocols mentioned BTS protein concentrations of “approximately” 40 mg/mL. However, they also acknowledged batch-to-batch variations and differences arising from using various species or rat strains. Over time, not only did the % v/v concentrations in the Ames protocols vary (ranging from 4 to 30%, see SM, Table S10), but discrepancies also appeared between these protocols and the foundation publications they were based on.^89,90^ This inconsistency underlines the methodological variations within this body of literature. Lastly, even the approximated 40 mg/mL BTS concentration cannot be used as a rough standard since we have encountered literature that employs the Ames protocols for BTS production but states divergent BTS protein concentrations (e.g., refs 91–93 ). While the historical Ames protocols focused on activity normalization using the number of revertant colonies in response to a reference control, such as aflatoxin B1, this method is not universally applicable to other BTS-relevant end points. Therefore, many studies referencing Ames et al. for BTS-related methodologies fail to specify critical parameters, rendering this approach inadequate for precise BTS characterization due to the inherent parameter variability and the evolution of these protocols over time.

Expert panel, best practice reports on the methodological implementation of the *in vitro* Comet Assay,^79^ and the *in vitro* micronucleus assay^80^ maintain the use of % v/v metrics, whereas the guidance for the chromosomal aberration assay^78^ recommends presenting BTS protein concentrations in mg/mL. Similarly, respective and more recent *in vitro* mutagenicity and genotoxicity-related OECD test guidelines (TG nos. 471, 473, 476, and 487;^15,16,39,40^ ) also do not specify reporting BTS concentrations in mg/mL. While all TGs demand defining S9 BTS type and composition within the TG test report, including the final S9 concentration in culture medium, they abstain from defining concentration metrics. Contextually, all of the named TGs address S9 concentrations in % v/v. Regulatory authority guidance relating to standardized *in vitro* genotoxicity tests (TGs), such as by the UK government C*ommittee on the mutagenicity of chemicals in food, consumer products and the environment* (COM), mentions the utilization of S9 BTS but does not give details on practical utilization or reporting standards.^94,95^ Finally, a recent volume of *Methods in Molecular Biology* published a series of protocols related to *in vitro* genotoxicity and mutagenicity.^96^ While many protocols define the final BTS reaction mixtures in detail regarding the employed cofactors, regeneration systems, and buffers, all rely on the % v/v for S9 BTS. Adherence to Ames’ historical protocols, and thereby the % v/v metric, may influence the methodological robustness in studies of mutagenicity and genotoxicity.

Another area for improvement is the “BTS dilution” subdomain. Although most records scored well (89% relative score; see SM, Table S6), some publications either did not define (not defined—“nd”) or were unclear (not clear—“nc”) in describing the dilution steps from BTS and cofactor stocks to final BTS reaction mixture concentrations. This lack of clarity means that the experimental setup is not reproducible; however, this issue can be easily rectified.

To conclude, it is essential to always define BTS protein concentrations in milligrams per milliliter and explicitly state the concentrations of BTS and cofactors at the stock and final reaction stages to ensure methodological robustness and reproducibility.

### 4.3 “BTS Characterization”—The Primary Culprit

In Section 3.2, we highlighted the primary data item domain “BTS characterization” as exhibiting the lowest methodological reporting soundness in our study, particularly in the subdomains “strain”, “pooling” (sex and number of individuals), “husbandry”, and “BTS induction” (see SM, Table S5). The iterative mapping analyses revealed a troubling trend: Negative reporting robustness patterns associated with externally purchased BTS, especially those derived from rats and used in conjunction with eukaryotic test systems and cell culture mediums (Figure 8). A notable increase in the use of externally sourced BTS over the years has been accompanied by a decline in the quality of BTS characterization reporting (see the SM, Figures S8C, S11C, S12C, and S14C). In contrast, BTS produced in-house consistently showed higher standards of reporting (Figure 9C). Per se, this is self-evident, given that experimenters who hand-craft the BTS are likelier to report on the specific parameter of BTS characterization. On the other hand, purchasing BTS from an external supplier was associated with noncompliance with reporting standards and a lack of certification/documentation from the suppliers. Dependence upon the mention of a BTS supplier or producer is insufficient, as it withholds specific details necessary for interpretation.

The rising trend in sourcing BTS externally is concerning, considering the critical role of BTS in high-throughput applications such as eukaryotic reporter systems. These applications often involve studying various toxicity pathways’ modes of action, where robustness in BTS characterization is crucial. To address this, we compiled a nonexhaustive list of BTS characterization items from different (anonymized) producers encountered within the investigated literature (see the SM, Table S12). Our findings indicate inconsistent BTS characterization among producers, particularly with respect to the assessment of phase 1 and 2 capacities. Producers offer a variety of BTS, differing in type (S9 or microsomes), species of origin, pooling, and chemical induction. The lack of standardization in BTS production protocols further complicates this issue, particularly where production has been discontinued, making it impossible to request or obtain any documentation. A standardized BTS production protocol is needed to guide production and ensure consistent reporting. Producers are encouraged to consider offering well-characterized BTS to meet these quality requirements, and the end user can promote this process by requesting such information from their supplier.

In practical applications, mainly when BTS is used in conjunction with pro- and eukaryotic test systems, the BTS protein concentrations typically range between 0.01 and 0.1 mg/mL in the final BTS reaction mixture (i.e., in the well of a microtiter plate). Various studies have identified concentrations within this range as optimal.^85,97–101^ Higher concentrations have been linked to cytotoxic effects or the introduction of BTS-derived cellular debris that could interfere with later-stage assay readouts, such as luminescence, fluorescence, or absorbance readings^102^ (Lungu-Mitea et al., in preparation). Thus, a small initial aliquot of well-characterized batches of BTS (5–10 mL) could suffice for an entire project. Ideally, producers could conduct characterization in a high-throughput manner during production, ensuring economic efficiency. This approach could align with the research and regulatory guidance framework discussed in Section 5.

### 4.4 Possible Solutions and a Way Forward

In contrast to the challenges highlighted in the previous sections, our analyses identified good reporting robustness patterns where the literature studies focused on metabolites and xenobiotic metabolism functionality as end points (Figures 7C, 8, and 9B). These studies predominantly utilized BTS in generic phosphate buffers (Figures 8 and 9G) and employed noninduced BTS (Figures 6F, 8, and 9F), usually of nonrodent origin, such as human or fish. This trend suggests that studies specifically targeting xenobiotic metabolism functionality are more likely to accurately address all BTS components owing to the precise focus of their objectives. Similarly, studies involving metabolite characterization post-BTS incubation, e.g., via mass spectrometry, often employing simpler BTS systems, also tend to report all relevant BTS constituents more consistently.

An intriguing robustness pattern emerged with the use of *in vitro*-derived BTS (Figures 6A and 9E), where BTS is extracted from immortalized hepatocyte cell cultures rather than animal-derived liver homogenates (e.g., refs 103–105 ). This approach is particularly noteworthy, as it aligns with the principles of reducing, refining, and replacing animal experiments. However, only a limited number of publications (*n* = 5) have adopted this method, suggesting that it is an emerging aspect but also highlighting the potential for significant growth and improvement in this area.

Moreover, the source species of BTS substantially influenced the quality of reporting (Figures 6C, 8, and 9D). Human-, mouse-, and fish-derived BTS generally received higher scores than rat-derived BTS. Human- and fish-derived BTS outperformed rat-derived BTS in overall scoring. Also, the comparison of fish-derived BTS to the rest of the data set indicated greater reporting quality (see SM, Figure S26).

Human-derived BTS is often more thoroughly reported, likely due to its status as a valuable pharmaceutical resource obtained from donor organs, coupled with the inherent variability in human phase 1 and 2 enzyme activity.^19,106^ However, the ethics in using human tissue for commercially related activities and the high variability reduce acceptability when it is used for chemical regulatory purposes at the global level. While in pharma, it is useful to have this variability, for chemical hazard assessment test methods, reproducibility is essential for regulatory acceptance. Indeed, the issues discussed around human serum are equally applicable to human BTS.^107^

As mentioned earlier, the contemporary ICH M12 Guideline recommends using hepatic *in vitro* systems and PBPK *in silico* modeling for drug interaction studies.^19^ It specifies using human liver tissue fractions, such as microsomal systems from at least 10 donors. Microsomal protein concentrations should be minimized, and standardized assay conditions, including buffer strength and pH, must be applied. BTS should be characterized with selective *in vitro* probe substrates for phenotyping experiments to confirm enzyme activity. It also lists probe substrates with marker reactions and advises inclusion of inhibitors or inducers as positive controls. Our analysis aligns closely with and complements the ICH M12 guidance, offering potential synergies for the scientific and regulatory communities.

Fish-derived BTS, typically from species like trout and carp, are rarely sourced externally and are usually prepared following established protocols,^108^ which have been adopted into OECD TG 319B.^109^ This guideline details essential steps and reporting recommendations, thereby inherently promoting methodological rigor in the research that applies them.

In contrast, “classical” BTS (male rat, Sprague–Dawley strain-derived, Aroclor or BNF/PB-induced, co-utilized with bacterial or eukaryotic test systems, buffered in culture medium, with exposure chemicals solved in DMSO or aqueous solutions, employing solely “w/o BTS” as a control, and investigating the end points mutagenicity, genotoxicity, and endocrine disruption) were associated with methodological reporting limitations in at least one analysis conducted in outcome C. Given these findings, we propose the development of a research and regulatory guidance framework applicable to all BTS types. Such a framework could enhance the standardization of BTS characterization and reporting, potentially integrating it into broader *in vitro* toxicology guidance, such as the GIVIMP^110^ and the Guidance Document for Describing Non-Guideline In Vitro Test Methods.^111^

## 5 Guidance Framework to Support Regulatory-Relevant and Research Applications (Outcome D)

Building upon the insights developed from the critical analyses of our results, synthesis, and discussion, we propose an evidence-based reporting guidance framework for external biotransformation systems (“BTS”, S9 or microsomal liver fractions), which is particularly relevant for experimenters employing BTS in conjunction with pro- or eukaryotic test systems such that their work can achieve greater utility. This framework aims to enhance methodological rigor and augment the value of data sets for subsequent analyses. The framework centers around key recording categories or data item (sub)­domains that scientific publications and reports should routinely include. By following these recommendations, researchers can contribute to a more robust and better utilized body of scientific literature. The guidance is intended to be self-reliant, allowing the reader to use it without familiarizing themselves with the preceding article. However, in some instances, referencing other literature or sections of the main manuscript is necessary to link to additional details, while ensuring coherence and conciseness.

Our guidance takes inspiration from existing protocols for fish-derived BTS, which our study identified as one of the most methodologically sound systems,^108,109^ combined with additional criteria derived from our analyses, together with the M12 guideline from the International Council for Harmonisation of Technical Requirements for Pharmaceuticals for Human Use (ICH).^19^ This approach also considers the challenges and issues previously discussed in the literature regarding the use of external BTS in toxicological *in vitro* systems.^6,7,9,21^ We provide a roadmap to help researchers navigate these challenges more effectively.

The proposed reporting categories are supplemented with brief discussions elucidating the scientific rationale behind their necessity. A separate reporting checklist is available as a specific, single supplement provided with the main article, which can be used to develop suitable study designs and facilitate the preparation of reports for publication. The reporting categories essentially mirror the data item domains extracted in our study, focusing on reliability aspects. If these data requirements are routinely included in scientific publications and regulatory study reports using BTS, then a concomitant improvement in the quality of BTS-related research is expected.

### BTS Characterization

#### I Determine BTS Origin (Internally Produced or Externally Purchased)

If produced in-house, define the production protocol in detail, including husbandry, organism source, organism supplier, housing conditions, organism weight, the weight of extracted liver tissue, gonadosomatic index, etc. Detailed BTS protocoling and reporting instructions for in-house production can be found in TG 319B and associated publications pertaining to trout S9.^108,109^ All of the following points (I.A to I.E) should be addressed when reporting on in-house-produced and applied BTS. If externally purchased, name the producer and give as many details as possible (aligned with points I.A to I.E). Ideally, this information is requested from the supplier prior to purchase. Until a common standardization of BTS production has been agreed upon and also implemented for BTS suppliers, we strongly recommend that investigators document all details of BTS characterization provided by the producer/supplier.

#### I.A Define the Type of BTS, e.g., Microsomal or S9

Defining the type and source of BTS is crucial, as microsomes and S9 fractions have different biotransformation capacities. While S9 BTS incorporate both mitochondrial and cytosolic fractions of the hepatic homogenate and are capable of both phase 1 and phase 2 biotransformation processes if supplied with the specific cofactors (see more details in points II.D and II.E), microsomes contain only mitochondrial fractions. Thus, they are limited to phase 1 metabolism and UDP-glucuronosyltransferase (UGT, phase 2) activities. Notably, even when investigating identical phase 1 processes, differences have been encountered between microsomal and S9 BTS.^112^

Given the scenario in which BTS are *in vitro*-derived (e.g., refs 104,105 ), it is essential to specify the cell culture origin and its biotransformation capacities, as some metabolism pathways may be limited in permanent or recombinant cell lines or by specific induction patterns sought and applied.^103,113^

#### I.B Define the Species of BTS Origin

Species of origin plays a pivotal role in the functionality of BTS due to varying biotransformation capacities and pathways, as extensively reported in the literature.^114–117^ Thus, specifying the species from which the BTS is derived is essential.

#### I.C Define the Strain of the Above Species, if Applicable

Strain-specific differences in rodent biotransformation capacities have been well-documented.^118–122^

#### I.D Provide Details on BTS Pooling Procedures, Especially the Number of Individuals and Sex

In humans, high variability within CYP isoforms has led to pooling across several individuals to reduce variability.^106,123^ Sex differences in rodent BTS production have been reported to impact biotransformation capacities on several occasions.^124–126^ Moreover, the biopsy location within the liver of the same individual used for BTS production can lead to variations in biotransformation capacity, highlighting the need for detailed specification^119^ and, eventually, pooling. The ICH M12 guideline^19^ specifies that a pool of at least 10 donors is preferred for human liver microsomes.

#### I.E Define the Biotransformation-Inducing Chemical Agents, if any, that Were Used. Explicitly State if No Chemical Induction Was Performed to Avoid Confusion

Studies have shown that different chemical inducers in source animals can lead to varying BTS capacities.^127–129^ With the Stockholm Convention,^30^ for OECD TGs, PCB-induced rat livers (e.g., via Aroclor) have been phased out in favor of phenobarbital plus beta-naphthoflavone mixtures (PB/BNF). PB/BNF induction has been described as qualitatively similar to Aroclor but with quantitative differences.^115,130,131^ Furthermore, “Aroclor” does not refer to a uniform mixture. Instead, it can encompass diverse profiles of PCB, PCDD, and PCDF congeners.^132^

### BTS Reaction Components

#### II Clearly Define All Reaction Components and Their Respective Concentrations within the Final BTS Reaction Mixture

The final BTS reaction mixture constitutes S9 or microsomal fractions, cofactors, buffers, solvents, and exposure chemicals (see points II.A-D below) after their addition to the employed *in vitro* assay and/or chemical exposure within the reaction vessel (microtiter well, cuvette, etc.) at the time point when all reaction components are simultaneously present and the biotransformation reaction starts (often also referred to as “incubation”, see also point III.A). Also, defining components’ concentrations within stocks and master mixes (dependent on the utilized test setup) is advised. For recommended concentration units, refer to points II.C and II.D. Finally, clearly marking the dilution/titration steps between stocks, master mixes, and the final BTS reaction mixture facilitates the reproducibility and clarity of the experimental protocol and is therefore recommended.

#### II.A Define the BTS Buffer System in which the Final BTS Reaction Takes Place

The buffer system in which the final BTS reaction occurs is critical, especially for cell culture medium-buffered systems. Different components in media, such as amino acids, vitamins, and cofactors, as well as pH, can impact BTS activity.^133,134^

#### II.B Specify the Utilized Solvent(s) for Chemical Exposure, Especially the Solvent Concentrations within the Final BTS Reaction Mixture

Several publications indicated that various organic solvents could impact phase 1 and phase 2 BTS enzymatic activity if utilized within the same reaction mixture.^73,86,135^

#### II.C Provide the S9 or Microsomal Fraction Protein Concentration in mg/mL, Not Percentages

Most importantly, provide the S9 or microsomal fraction protein concentration in the final BTS reaction mixture. S9 or microsomal fraction protein concentration in mg/mL can be utilized to normalize the results of downstream readouts related to BTS biotransformation processes. Reporting BTS protein concentrations in mg/mL has been independently suggested by different authors (e.g., refs 21,85,86 ). Furthermore, defining the BTS protein concentration can give the reader additional insight into the potential cytotoxic effects of BTS.^87,88^

#### II.D Name the Utilized Cofactors Appropriately and Define Their Molar Concentrations at Least within the Final BTS Reaction Mixture

Familiarize yourself with the cofactors needed for your research question. If a NADPH regeneration system is utilized, define all components fully. Optimal use of cofactor combinations, regarding BTS utilization in a specific experimental setup, and NADPH regeneration systems are discussed in previous studies (e.g., refs 70,71,73,74 ). Characterizing the utilized regeneration systems is vital, as different systems can lead to varying results.^71,136^ It is also noted that cofactors and regeneration systems can introduce cytotoxicity themselves in specific scenarios.^137^

#### II.E Define, Report, or Measure BTS Enzymatic Activity

Lack of standardization in assessing BTS-induced enzymatic biotransformation activity necessitates obtaining the producer’s activity information as part of the Certificate of Analysis or determining it independently if not forthcoming from the supplier. For now, when purchasing external BTS, we recommend indicating all information given by the producers and requesting missing key information. If information on BTS enzymatic activity cannot be obtained from the producer or vendor or for in-house produced BTS, conduct the measurement yourself (e.g., CYP1A, CYP3A, CYP2B6, CYP2C9, and GST). Suggestions on enzymatic activity end points and protocols can be found in the literature.^19,86,138,139^

For tiered testing strategy approaches that require preliminary screening and studies investigating toxicological mechanisms of action, e.g., via eukaryotic test systems complemented with BTS, it is recommended to report the relevant information obtained from the supplier documentation, where available, or, if not available, to independently determine the enzymatic activity of all respectively studied phase 1 and phase 2 reactions for which specific cofactors have been added to the BTS. For regulatory information requirements or in a best-case scenario, it is recommended to characterize all enzymatic biotransformation activities (CYPs, GST, UGT, and SULT activities). These can be assessed, e.g., via HPLC MS-MS^17,140^ or metabolic turnover of fluorometric substrates.^141^ Notably, a live-cell, fluorometric, enzymatic activity assessment battery protocol for these end points is underway (Lungu-Mitea et al., in preparation).

### BTS Experimental Setup

#### III.A Define BTS Incubation Time and Temperature

Incubation time must be considered in relation to the employed BTS protein and cofactor concentrations so that biotransformation reactions can realistically proceed.^21^ Studies employing prolonged incubation must assess potential cytotoxicity impacts.^142^ If nonmammalian BTS are applied, the incubation temperature should be adjusted accordingly.^112^ Optimally, experimenters are advised to also report on the circumstances of incubation, including the use of shaking devices and the type of reaction vessel (glass, plastic, etc.).

#### III.B Define BTS-Related Controls

Carefully select a set of BTS-related controls according to the study design and objectives; their proper implementation and documentation are necessary for any BTS applications. Appropriate BTS controls are vital for interpreting the impact of the chemical biotransformation on its bioactivity, distinguishing between systems with induced biotransformation,^78–80^ assessing self-sustained biotransformation potential,^81^ and evaluating the impact of BTS on the bioavailability of chemicals.^82,83^ For studies that employ the BTS alongside pro- or eukaryotic test systems, we recommend using at least the “w/o BTS” (without BTS and without cofactors, per se the plain *in vitro* assay) and heat or chemically “inactivated BTS” (excluding cofactors) controls to evaluate the biotransformation impact and address bioavailability-related issues, respectively. More detailed reasoning on the necessity of these BTS-related controls is given in Section 4.1 of the main article and Table S11 in the SM. Additional BTS control scenarios might be necessary or informative when developing, characterizing, and troubleshooting novel methods, as well as for specific BTS applications, which is also addressed in Table S11.

## Supplementary Material





## Data Availability

All data generated or analyzed throughout this study are included in this article (and its supplementary files; the supplementary manuscript (SM), supplementary information material files (SI1−S11), and the reporting checklist). The SM provides in-depth documentation of the utilized systematic tools, illustrates additional data, and discusses the conceptual framework of the review. Additional supplementary information material files comprising raw data, metadata, and analyses have been uploaded to Figshare and are available under the following link: Figshare (10.6084/m9.figshare.30257470,^55^ *accessed 2025/10/17*). Eleven supplementary information material files (SI1−SI11) include: SI1, complete data set for endocrine disruption end point searches (“BTS1” data set); SI2, complete data set for mutagenicity and genotoxicity end point searches (“BTS2” data set); SI3, data set of endocrine disruption end point screening selections; SI4, data set of mutagenicity and genotoxicity end point screening selections; SI5, bibliographic records previously identified as relevant by reviewers and associated cross-references (“BTS3” data set), including their respective screening selections; SI6, comprehensive data set of all extracted quantitative and qualitative data item measures across (sub)­domains, forming the foundation for all subsequent meta-analyses; SI7, DEERS assessment-derived total scores; SI8, raw and meta-level data from meta-regression analyses; SI9, coding simplifications of data item measures (from SI6) for machine readability, including a binary information matrix for multiple correspondence analysis; SI10, additional association networks (100 to 5000 rules) generated using Apriori algorithms; and SI11, raw and meta-level data from follow-up, confirmatory analyses.

## Notes

BTS reporting checklist: a concise, user-friendly checklist for BTS methodological reporting (PDF)
The Supplementary Manuscript (SM): detailed conducted methodologies, additional results and discussion, references of all bibliographic records, R-code, DEERS data extraction form, and an abbreviation list (PDF)
Additional supplementary information material files (SI1−SI11) are described in the Data Availability Statement section above.
