# Supplementary material for: On the Utilization and Characterization of External Biotransformation Systems in In Vitro Toxicology: A Critical Review of the Scientific Literature with Guidance Recommendations
Source: ACS Environ Au. 2025 Nov 27;6(1):21–45. doi: 10.1021/acsenvironau.5c00096 (PMC12828618; doi:10.1021/acsenvironau.5c00096)
Supplement: Supplementary file 1 [file vg5c00096_si_001.pdf]

BTS reporting checklist	
Reporting category	Check/details
**BTS characterisation**	
I. Determine BTS origin (internally produced or externally purchased).	
I. A) Define the type of BTS, e.g., microsomal or S9.	
I. B) Define the species of BTS origin.	
I. C) Define the strain of the above species, if applicable.	
I. D) Provide details on BTS pooling procedures (number of individuals and sex).	
I. E) Define the biotransformation-inducing chemical agents. State if no chemical induction was performed.	
**BTS reaction components**	
II. Clearly define all reaction components and their respective concentrations within the "final BTS reaction mixture".	
II. A) Define the BTS buffer system in which the final BTS reaction takes place.	
II. B) Specify the utilised solvent(s) for chemical exposure, especially the solvent concentrations within the "final BTS reaction mixture".	
II. C) Provide the S9 or microsomal fraction protein concentration in mg/mL, not percentages.	
II. D) Name the utilised cofactors and define their molar concentrations.	
II. E) Define, report, or measure BTS enzymatic activity.	
**BTS experimental setup**	
III. A) Define BTS incubation time and temperature.	
III. B) Define BTS-related controls.	
